# Proteogenomic analysis of *Serratia marcescens* using computational subtractive genomics approach

**DOI:** 10.1371/journal.pone.0283993

**Published:** 2023-04-10

**Authors:** Sharon Elaine D’Souza, Kanwal Khan, Reaz Uddin

**Affiliations:** Dr. Panjwani Center for Molecular Medicine and Drug Research, International Center for Chemical and Biological Sciences, University of Karachi, Karachi, Pakistan; Nazarbayev University School of Medicine, PAKISTAN

## Abstract

*Serratia marcescens*, a Gram-negative bacterium (Enterobacteriaceae) is a hospital-acquired opportunistic pathogen that infects the urinary and central nervous systems. The identification of new therapeutics against *S*. *marcescens* is crucial since it is now multi-drug resistant. Therefore, the current study was aimed to identify potential drug targets against *S*. *marcescens* strains i.e. WW4, SM39, and Db11 using comparative metabolic pathway analysis and subtractive genomics approach. The applied bioinformatics-based method was used to identify the unique metabolic pathways as the prioritized drug targets. The downstream analysis has led to the identification of three pathways that are specifically absent and/or present in the specific strain. Consequently, six proteins were identified through subtractive genomic analysis. The identified proteins were found as non-homologous and essential to the pathogen’s survival as well as unique to the WW4 strain. The estimated features proposed it as a potential drug target. The selected protein was further subjected to in-depth structural analysis for the structure modeling, structure validation, and protein-protein interaction analysis. Furthermore, the library of ~1500 approved compounds was screened against selected drug target to identify potential drug candidates. The current work may help in repurposing of the drug compounds as novel medication against *S*. *marcescens*.

## 1. Introduction

*Serratia marcescens* is an opportunistic nosocomial pathogen and causative agent of many infections including urinary and central nervous systems (meningitis) as well as heart (endocarditis) and wound infections [1, 2]. *S*. *marcescens* affects immunocompromised patients specifically those undergoing broad-spectrum antibiotic therapy. The bacteria spread mostly through invasive instrumentation such as intubation material, intravenous and urinary catheters [3, 4]. The pathogenicity of *S*. *marcescens* can be attributed to several virulence factors such as the pore-forming toxin hemolysin, the serralysin protease or a phospholipase [5–7]. Presently, *S*. *marcescens* has been reported as an epidemic, primarily in The Neonatal Intensive Care Units (NICUs) and Intensive Care Units (ICUs) [8, 9]. *S*. *marcescens* are now generally multidrug-resistant [10].

Currently, antibiotic resistance is a threat to antibacterial therapies [11]. The antimicrobial resistance posed a serious threat to treat a pathogen because of the limited options. During the last twenty years, *S*. *marcescens* was empirically treated using aminoglycoside, piperacillin-tazobactam, carbapenem, or fluoroquinolone. Later, modification of the treatment was suggested based on the strain susceptibility test results [1]. A recent study revealed that *S*. *marcescens* is intrinsically polymyxin resistant which is an antibiotic being used as a last resort to treat carbapenem-resistant *Enterobacteriaceae* [12]. The WHO has classified species of *Serratia* under the critical category as they are resistant to carbapenem [13]. Research into the antimicrobial resistance genes among the *S*. *marcescens* using public whole-genome datasets helped in the detection of 100 AMR (antimicrobial resistant) genotypes. Aminoglycoside and beta-lactam resistant genes including ESBL (Extended-spectrum beta-lactamases) and carbapenemase genes are highly prevalent in clinical isolates of *S*. *marcescens* [14]. The limited research against hospital-acquired infections during last decade has translated into a deficit of drugs against multidrug-resistant pathogens [15, 16]. There is a dire need of innovative therapeutic interventions against resistant bacteria [17]. There has been an increased demand of research on *S*. *marcescens* in recent times due to its antibiotic resistance capability [18].

Different computational methods have been used earlier to search new chemical entities as potential drug candidates. In this context, comparative subtractive genomics coupled with metabolic pathway analysis produced robust data to prioritize unique essential proteins that may serve as drug targets. These essential proteins may act as putative targets for designing novel drugs. Previously, numerous such applications are reported in literature to propose new drug targets against deadly pathogens [19]. Contrary to the traditional drug discovery process which is tedious, time-consuming and expensive, computational discovery of drug targets is a rational approach. It speeds up drug discovery, expands therapeutic options, and decreases rate of failure of drugs in clinical trials. Bioinformatics methods can predict drug targets based on its selectivity/specificity and essentiality, cellular function, location inside the cell, the ability to be targeted by broad-spectrum agents, its functional interactions with proteins of metabolic pathways and druggability [20].

Even though *S*. *marcescens* has been extensively studied to identify potential drug targets yet the current applied method has not been reported to our knowledge. Furthermore, this work will help to identify potential drug targets.

## 2. Material and methods

**Fig 1:**
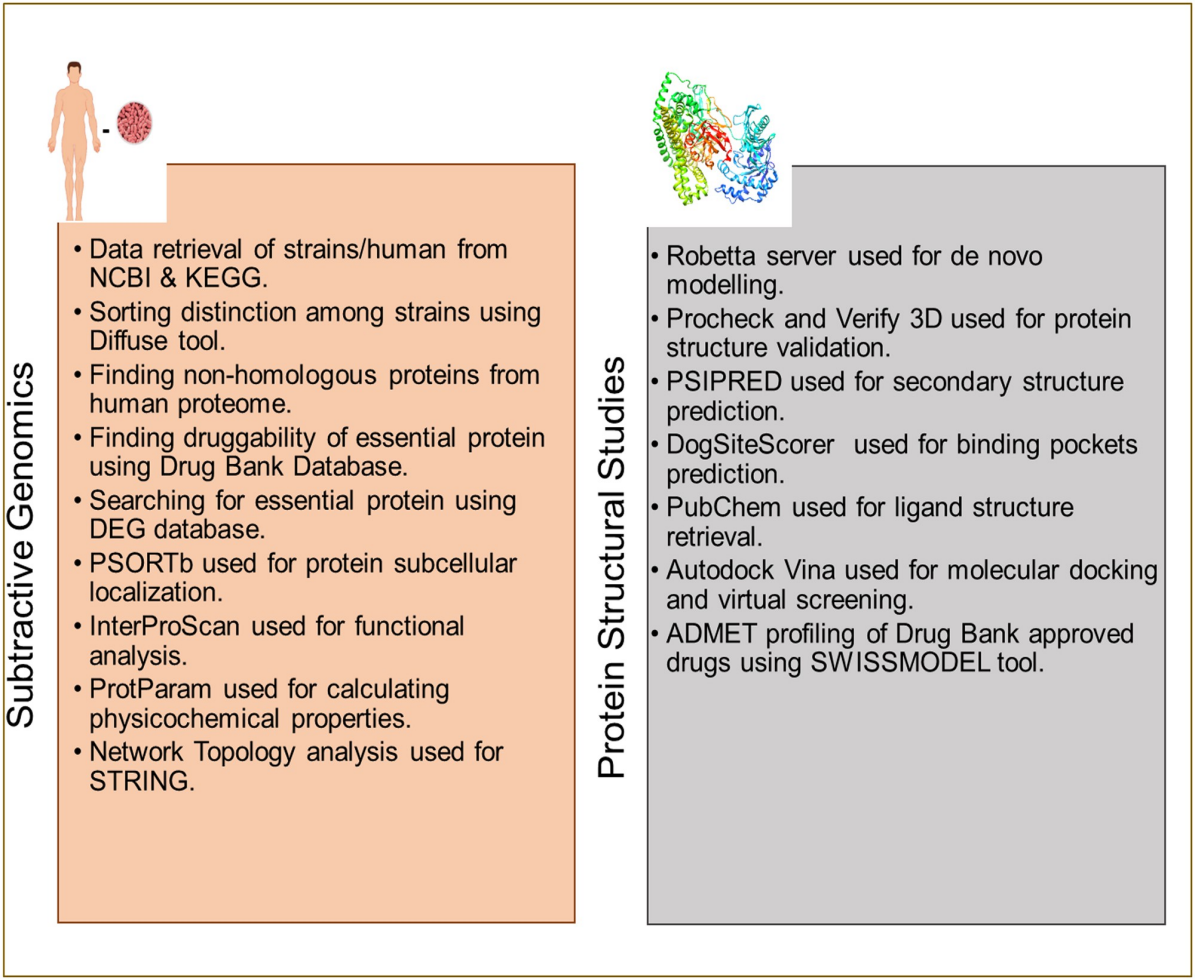
Flow chart of applied methodology in current study. Potential drug targets against *S*. *marcescens* identified using this methodology.

In this study, we used all available strains of *S*. *marcescens* as part of the analysis. The strains were analyzed to find potential drug targets retrieved from metabolic pathways of the pathogen. Metabolic pathways are a network of interconnected metabolic events that occur in a cell. Some of these metabolic pathways and metabolites are required essentially by the pathogens to survive. Therefore, it is prudent to target such metabolic pathways to eradicate the pathogen. Thus, the metabolic data of three available *S*. *marcescens* strains were retrieved. Metabolic pathways unique to the pathogen compared with *Homo sapiens* were selected using metabolic data. Essential proteins of these metabolic pathways were shortlisted using BLAST against the DEG database. Moreover, protein structural studies were conducted to model a 3D structure and locate the binding pockets of the protein. Finally, protein characterization and network topological analyses were performed. The current study is divided into two phases i.e. (1) Subtractive Genomics and (2) Structural Studies. The details of the study are provided in the workflow (Fig 1).

### 2.1. Phase I: Subtractive genomics

#### 2.1.1. Data retrieval

Databases including NCBI [21] and KEGG [22] were used to retrieve data on three specific strains of *S*. *marcescens*. Dataset of the human proteome was retrieved from the NCBI. The data of the three available strains were accessed on 10th Feb 2022 from KEGG i.e., WW4, SM39, and Db11 strains. The htext files of the mentioned strains of *S*. *marcescens* were retrieved. Bash scripting was used to extract the metabolic pathway data of each strain from the BRITE hierarchy file. The script is provided in S1 Appendix. Table 1 contains information such as the organism’s name, its 3 lettered code and its respective T-number.

**Table 1:** Detailed information on different strains of *S*. *marcescens* used in current study.

Organism name	No. of Pathways	Estimated No. of Proteins	KEGG Code	T-Number
***Serratia marcescens*** SM39	110	2,967	smar	T03592
***Serratia marcescens*** subsp. marcescens Db11	112	2,976	smac	T03985
***Serratia marcescens*** WW4	111	2,985	smw	T02448

#### 2.1.2. Identifying differences in the strains

A tool known as Diffuse (http://diffuse.sourceforge.net/) was employed to sort the distinction among three strains graphically. Metabolic pathways that were uniquely present or absent among three pathogenic strains were visualized. Studies have reported the role of specific pathways and how their presence or absence impact the survival of the pathogen [23]. Further analysis was conducted on sequences of protein belonging to unique pathways, retrieved via KEGG. Proteins found in common metabolic pathways were discarded.

#### 2.1.3. Non-homologous protein identification

The shortlisted proteins of three strains from *S*. *marcescens* were subjected to a comparative analysis with the host’s human proteome using the BLASTp standalone tool [24]. Two inputs are required to execute the BLASTp namely (i) a sequences database and (ii) a query sequence with an optimum *e*-value such as 1e^-3^. The E value is the BLAST tool’s statistical parameter defining the expected maximum hits while making a comparison against a specific portion of the database. An inversely exponential relationship exists between the scores and the number of matches. Later on, “no hits” proteins against the human proteome were used for further downstream analysis.

#### 2.1.4. Druggability of non-homologous proteins

Additionally, the resulting nonhomologous proteins were assessed using BLASTp with an E value of 10^−5^ against the DrugBank database [25] to establish the druggability and find new drug targets. Proteins that showed the strong sequence similarity with the DrugBank version 5.1.9 All Drug Target library were inferred as potential drug targets, whereas those with no similarity were termed as novel drug targets.

#### 2.1.5. Identifying essential proteins

Annotation of the *S*. *marcescens* non-homologous proteins was performed using BLASTp. The Database of Essential Genes (DEG) helped in the identification of proteins essential to the survival of bacteria [26]. The BLASTp was performed for the identification of essential genes retrieved form the DEG prokaryotic data with a cut-off value of 1e^-5^, identity more than 30%, and query length more than 70% to identify essential proteins.

#### 2.1.6. Protein characterization

The characterization of protein’s subcellular location is a crucial aspect of its functional analysis. The PSORTb v 3.0 [27] predicted the subcellular location of the shortlisted proteins as it includes archaeal and bacterial diversified cellular morphologies. Thus, subcellular localization was performed on the identified drug targets. Furthermore, InterProScan analyzed the function of the protein and its annotation was performed using predictive signatures in the protein’s primary sequence [28].

#### 2.1.7. Physicochemical parameter analysis

The physicochemical properties of the selected proteins were estimated through ProtParam server [29]. The ProtParam computes the molecular weight, isoelectric point, atomic and amino acid composition, estimated half-life, coefficient of extinction, instability and aliphatic indices, and the protein’s Grand Average of Hydropathicity (GRAVY).

#### 2.1.8. Protein interactome analysis

The protein interaction network analysis for the pigC protein known as prodigiosin synthetase that is involved in the synthesis of the antimicrobial compound prodigiosin was conducted *via* a popular tool i.e., STRING [30]. It predicts a protein’s functional and physical associations. Data from various sources are integrated statistically for a wide number of species, STRING transmits information between these organisms. At the time of this study, there are 5,214,234 proteins from 1133 species in the database. In this study, the protein-protein interaction of the ’essential’ cytoplasmic proteins was performed using this tool.

### 2.2. Phase II—Protein structural studies

BLAST was implemented online against PDB using NCBI [31] to predict an appropriate template for the structural modeling of the selected protein. The protein homology modeling was performed using the RoseTTA fold method available on the Robetta webserver [32]. Additionally, ProCheck [33] and Verify3D [34] were used to validate the structure of the modeled proteins. PSIPRED [35] predicted the protein’s secondary structure.

#### 2.2.1. Docking of ligands

Moreover, DogSite Scorer was used to find protein binding pockets [36]. This uses statistical analysis to rank the binding site. The binding site with the highest score was used in the study. The binding ligands for the shortlisted protein were retrieved from the KEGG database. The KEGG database showed that our shortlisted protein binds with three ligands. The structures of these ligands were retrieved from PubChem [37]. Moreover, Autodock [38] was used for the docking of these ligands using the selected binding pocket in the individual sessions. In all sessions, the number of Genetic Algorithm Parameters (GA) was 50 runs, and the population size was 300 to ensure reasonable docking results. Other parameters, which included the ga_num_evals were selected in proportion to the number of torsions in the ligand. The docking conformation with the lowest binding energy was selected and the docked complex was visualized through Chimera [39]. Furthermore, Ligplot was also used to get an insight into the 2D interactions of the protein and ligand [40] in terms of hydrogen, and hydrophobic interactions.

#### 2.2.2. Virtual screening

~1500 compounds in the approved drugs library were retrieved from the Drug Bank database to screen against the shortlisted drug targets. The analysis of the structural error of all target proteins was performed using ADT (Auto Dock Tool). Grid box parameters and configuration files of the drug target were individually prepared using the above docking parameters. Configuration files for the target covering the active site of the protein predicted through DogSiteScorer were set as *N*º of points in X, Y, and Z-dimensions were 126 and Center Grid Box: X center: 21.294, Y center: 47.682, and Z center: 25.044, respectively to allow the molecular docking to occur in the selected binding pocket having the active residues retrieved from DogSiteScorer (LEU_182, THR_183, ALA_301, LYS_302, VAL_304). The AutoDock Vina was used to perform molecular docking.

#### 2.2.3. ADMET profiling

The chemical descriptors and druggability of compounds were analyzed using the SWISSADME tool. The database was used to determine the pharmacokinetics of the compounds such as absorption, metabolism, druggability, rule of five, intestinal absorption, Caco2 permeability, and toxicity [41]. Ames, carcinogenicity, hepatotoxicity, and skin sensation were applied as a toxicity parameter to screen endpoint models of the chosen compounds.

#### 2.2.4. Conservancy analyses of predicted sequences with other strains

The extent of the pharmacological spectrum over the whole homologous bacterial population may be inferred through comparison of the predicted sequences’ conservation pattern with other strains that are used conventionally. Therefore, the online BLAST [42] was used to conduct a conservancy analysis of the pigC protein. The BLAST was implemented using default settings with the exception of the restricted taxonomy option, where we specified *S*. *marcescens* with the taxonomic ID of 615 and UniProt KB was selected as the target database.

## 3. Results

### 3.1. Phase I: Subtractive genomics

#### 3.1.1. Potential drug target identification

After retrieving data of the metabolic pathways, the differences among the strains were annotated and identification of unique or absent pathways among the strains was inferred.

It was observed that the SM39 showed the greatest number of missing pathways and the Db11 showed the most additional number of pathways such as the Styrene and Polycyclic Aromatic Hydrocarbon Degradation pathways. The WW4 strain was found with an additional pathway unique to itself only i.e., the Prodigiosin Synthesis pathway.

**Fig 2:**
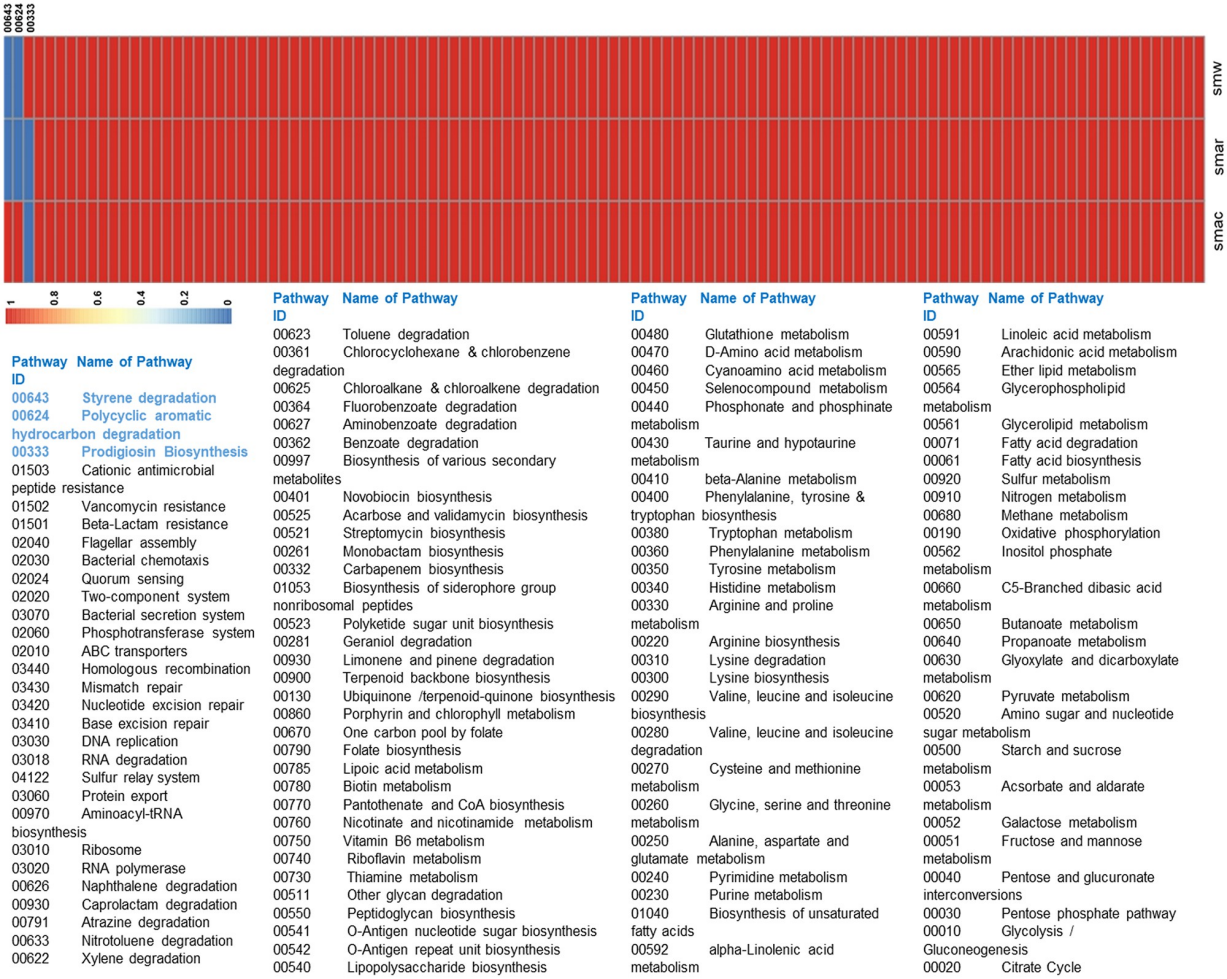
Heatmap showing the metabolic pathways in three strains of bacteria. Blue represents absence and brick-red represents presence of a pathway. The ids for the first three pathways have been provided in the heatmap with the remaining ids provided in the key. The unique pathways have been highlighted in blue in the key. The strains are denoted as smw (WW4), smar (SM39) and smac (Db11) strains according to Kyoto Encyclopedia of Genes and Genome (KEGG).

All pathways showed that they have played roles in the survival of bacteria with the Prodigiosin Synthesis pathway giving it a competitive edge over the other microbes. This compound was earlier reported to provide a survival advantage to bacteria against its competitors (other bacteria) and predators (nematodes and protozoans) as its secretion causes membrane disruption of other organisms [43]. A heatmap graphically representing the metabolic pathways present in the three strains of *S*. *marcescens* is shown (Fig 2). S1 Table shows the names and ids of these pathways as displayed in (Fig 2).

#### 3.1.2. Identifying non-homologous and essential proteins

The heatmap showed that the strains Db11 and WW4 were unique and hence subsequently used for further analysis. The proteins of those specific metabolic pathways were retrieved from the KEGG database. Among twenty proteins from both strains, two proteins were found in the Db11 strain and four in the WW4 strain that were non-homologous to the human proteome. BLASTp was then implemented against the DEG database using these six proteins, which resulted in only one protein i.e., the pigC protein as essential to the survival of the bacteria. The implemented BLASTp parameters and their respective outcomes have been mentioned in Table 2.

**Table 2:** Description of BLASTp parameters implemented and their predicted outcomes.

Program	BLAST+2.12.0	BLASTp of DEG	BLASTp of DrugBank
E value	1.00E-03	1.00E-05	1.00E-05
Query name	Unique/missing proteins	Non homologous proteins	Unique/missing proteins
No. of Queries	20	6	1
Name of Subject	Human proteome	DEG	All Drug Target

Details of these pathways and their proteins are mentioned Table 3. According to the literature survey, non-homologous proteins can serve as potential drug targets as they are unique to the pathogen thus turning them suitable as drug targets [44].

**Table 3:** List of 20 shortlisted proteins and their unique metabolic pathways.

Strain	NCBI-Protein ID	Metabolic Pathway	Proteins
**WW4**	AGE16905	Prodigiosin biosynthesis	3-acetyloctanal synthase
	AGE16904		3-acetyloctanal aminotransferase
	AGE16907		2-methyl-3-n-amyl-dihydropyrrolel dehydrogenase
	AGE16900		L-proline---[L-prolyl-carrier protein] ligase
	AGE16902		peptidyl carrier protein
	AGE16908		L-prolyl-PCP dehydrogenase
	AGE16899		beta-ketoacyl ACP synthase
	AGE16901		4-hydroxy-2,2’-bipyrrole-5-methanol synthase
	AGE16896		4-hydroxy-2,2’-bipyrrole-5-methanol dehydrogenase
	AGE16903		4-hydroxy-2,2’-bipyrrole-5-carbaldehyde O-methyltransferase
	AGE16906		prodigiosin synthetase
	AGE17665		[acyl-carrier-protein] S-malonyltransferase
	AGE17666		3-oxoacyl-[acyl-carrier protein] reductase
	AGE16379		3-oxoacyl-[acyl-carrier protein] reductase
	AGE18830		3-oxoacyl-[acyl-carrier protein] reductase
	AGE19637		3-oxoacyl-[acyl-carrier protein] reductase
	AGE20192		putative oxidoreductase
**DB11**	CDG13243	Polycyclic aromatic hydrocarbon degradation	3-oxoacyl-[acyl-carrier protein] reductase
	CDG10834	Styrene degradation	phenylacetaldehyde dehydrogenase
	CDG15262		catechol 2,3-dioxygenase

Proteins non-homologous to humans are been highlighted in grey. These non-homologous proteins were blast against the DEG database resulting in only one essential protein to the survival of bacteria i.e., prodigiosin synthetase.

#### 3.1.3. Druggability of non-homologous protein

BLASTp search against Drug Bank targets resulted in the identification of zero hits i.e., no hits were found using the set parameters for the pigC protein. Finding “No hits” does not necessarily mean that the protein cannot be used as a therapeutic target. However, the target was classified as novel target due to their absence in the Drug Bank database i.e., the protein in question could be a good target to be explored in further detail.

#### 3.1.4. Essential protein analysis

The main criterion that a potential drug target must satisfy is its essentiality for the survival of bacteria. Targeting such proteins may specifically kill bacteria. The essentiality of the non-homologous-druggable proteins was assessed by comparing them with the DEG database using BLASTp. This resulted in the identification of a single protein i.e, Prodigiosin synthetase as a novel drug target against *S*. *marcescens* that is vital for bacterial survival. The remaining five proteins without any hit were confirmed as non-essential and therefore, discarded. Hence, the identified essential protein was classified and selected as a novel drug target against *S*. *marcescens* i.e., prodigiosin synthetase.

#### 3.1.5. Significance of selected protein

The selected protein was annotated as Prodigiosin synthetase encoded by the gene *pigC* found in the WW4 strain of *S*. *marcescens*. This protein was found to be unique to the WW4 strain and is part of the accessory genome of the organism. The metabolic pathway in which it involved in is named Prodigiosin Biosynthesis (KEGG ID = 00333). A comprehensive literature review was conducted to find multiple roles of prodigiosin synthetase in pathogens and in other microbes.

In brief, this enzyme is involved in the prodigiosin biosynthesis i.e., Prodigiosin has anticancer, antimalarial, antifungal and antibacterial properties. As the pathogen invests a huge amount of energy into the biosynthesis of prodigiosin, it can be inferred that it is critical to the survival of *S*. *marcescens*, particularly during interspecies competition. Targeting such a protein will inhibit the essential pathway, inhibit the synthesis of this antimicrobial agent and making the bacteria susceptible to the treatment.

#### 3.1.6. Comparative protein analysis

Sequence alignment of the selected protein was performed with its homologs in different species to analyze its phylogenic relationship. The alignment was performed using Clustal Omega V.1.2.4. The protein sequence of the selected protein in *S*. *marcescens* was compared with homologous proteins in *Listeria monocytogenes* and *Neisseria meningitides* by Clustal Omega 1.2.4 [45]. The results showed very low similarity between the enzyme found in the *S*. *marcescens* and those of other bacterial species. The percentage similarity of the prodigiosin synthetase with different species is highlighted Table 4.

**Table 4:** Percent similarity matrix.

**Proteins**	**SMWW4_v1c11020**	**pdb|2OLS|A**	**pdb|5FBT|A**	**pdb|5FBS|A**	**pdb|5HV1|A**	**pdb|5HV2|A**	**pdb|5HV6|A**
**SMWW4_v1c11020**	100.00	25.33	26.33	26.85	26.81	26.81	32.51
**pdb|2OLS|A**	25.33	100.00	27.17	27.45	27.41	27.41	35.44
**pdb|5FBT|A**	26.33	27.17	100.00	97.12	97.12	97.00	97.78
**pdb|5FBS|A**	26.85	27.45	97.12	100.00	100.00	99.88	100.00
**pdb|5HV1|A**	26.81	27.41	97.12	100.00	100.00	98.75	96.94
**pdb|5HV2|A**	26.81	27.41	97.00	99.88	98.75	100.00	100.00
**pdb|5HV6|A**	32.51	35.44	97.78	100.00	96.94	100.00	100.00

Similarity matrix of *Serratia marcescens* associated homolog of prodigiosin synthetase with its partner protein in different species.

### 3.2. Phase II: Structure-based studies

**Fig 3:**
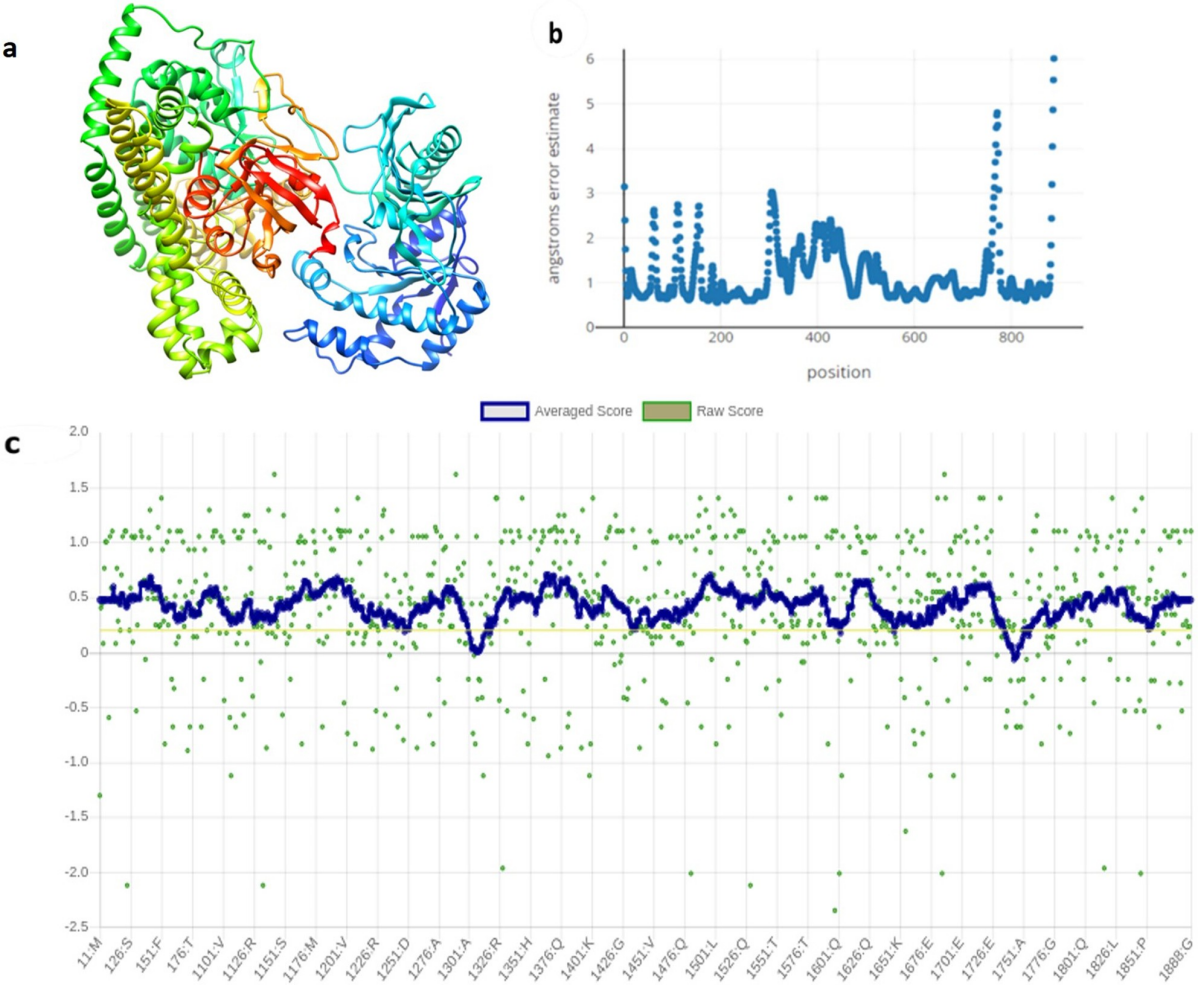
Results of protein structural based studies. **(a)** Modelled protein structure of prodigiosin synthetase through Robetta. **(b)** Angstrom Error Estimate graphically shown. **(c)** Verify 3D Plot showing the quality of modelled structure having 95.83% in allowed region.

De novo structural modeling was used as an alternative method in the absence of a suitable template by using the RoseTTA fold for the selected protein sequence. (Fig 3a) shows the modeled structure of the query protein, its angstrom error estimate, and the results of structural assessment studies. The results showed a confidence score of 0.84 with 1 being the highest.

The angstrom error estimate of the model showed an undulating pattern. The error estimate varied with 0.7 being the lowest and 6.02 is the highest, respectively (Fig 3b).

The modeled structure of the protein was evaluated using Verify 3D tool. This tool is used for the quantitative evaluation of the overall quality of the protein’s three-dimensional structure. Computationally, the modeled structure was classified with a quality score of 95.83% with at least 80% of the amino acids having a score of ≥ 0.2 (Fig 3c).

Additionally, the protein’s secondary structure was validated using PsiPred. It computationally estimates the secondary structural elements using the primary structure of the query protein. Yellow-colored bars represent strands while straight lines represent coils and pink colored bars represent helices. S1 Fig shows the PsiPred results predicting the position of strand, helices and coils. The earliest strand spans from residues 6–8 while the first helix spans from the residues 17–19. A comparison was made between the PsiPred results and the modeled three-dimensional structure, which revealed that the results mostly aligned with the modeled structure of the protein.

The Ramachandran plot was generated for the modeled protein to evaluate its quality. It showed that only 0.6% of the residues were found in the disallowed region. The ProCheck classified 91.5% residues in the core allowed region whereas 7.5% in the allowed and 0.4% in the generously allowed region. The Ramachandran Plot showed that five residues fall in disallowed region inclusive of asparagine, lysine, isoleucine and two serine residues, while showing the overall good quality of the structure as shown in S2 Fig.

#### 3.2.1. Protein characterization

The selected protein was characterized by studying its subcellular location, physicochemical characteristics, superfamily and interaction network. Details are provided in the following sections:

#### 3.2.2. Sub–cellular localization prediction and functional family classifications

**Fig 4:**
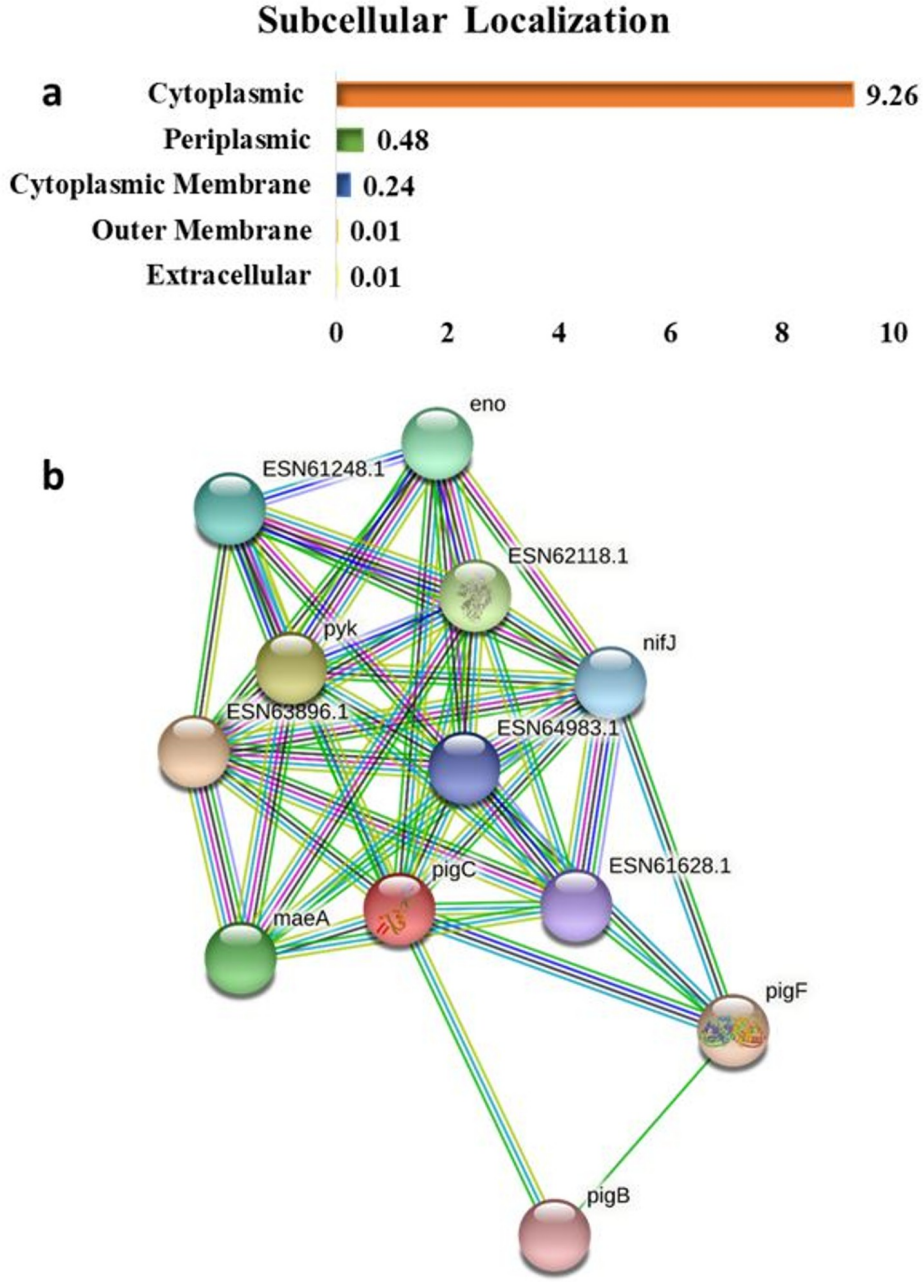
Functional characterization of protein. **(a)** The sub-cellular localization of protein. The results are indicating that the query protein belongs to cytoplasmic region in the cell. **(b)** Image showing the network topology of the query protein in red color (PigC) generated by STRING database showing the top 11 interactions.

The identification of protein sub-cellular localization is important to effectively characterize the protein’s function and chemical nature. Additionally, it aids in the evaluation of the molecular structure and druggability of the protein. Consequently, the selected protein was classified as cytoplasmic as 9.26% was computed as cytoplasmic while 0.24% was found in the cytoplasmic membrane. The bar chart presented in (Fig 4a) shows the protein’s sub-cellular localization distribution. It was observed that the identified drug target was located within the cytoplasmic region of the pathogen.

The InterProScan is an online tool that computes a protein’s functional domains and predicts its super-family which function as diagnostic signatures for the classification of the proteins. The InterProScan predicted the protein’s ATP binding functional domain. This prediction serves as a diagnostic marker about the functional domain and the type of compounds it interacts with. This tool predicts that the protein is associated with the phosphorylation pathway within the cell. S3 Fig shows the InterProScan results.

#### 3.2.3. Physicochemical properties prediction and analysis of network topology

The ProtParam computed the protein’s physicochemical properties. The Gravy index was estimated at -0.197 inferring that the drug target is hydrophilic. The theoretical *p*I of the protein was 6.45 thus implying that the protein is acidic. As the protein’s instability index is higher than 40, the protein is considered unstable. Table 5 shows the ProtParam results.

**Table 5:** Physicochemical properties of prodigiosin synthetase.

**Physicochemical Properties**	
Number of amino acids	888
Molecular weight	99267.56
Theoretical pI	6.45
Instability Index	43.70
Aliphatic Index	88.41
Grand average of hydropathicity (GRAVY)	-0.197
**Estimated half life**	
Mammalian reticulocytes, In-vitro	30 hours
Yeast in-vivo	>20 hours
*Escherichia coli*, in-vivo	>10 hours

Centrality is one of the many characteristics a drug target has. Additionally, a proposed drug target has a hub and central role in the protein network. The STRING database predicted the associated network of the prioritized protein. About 198 nodes are associated with the prioritized protein confirming its hub role within the network. Detailed scores of interacting nodes are provided in S2 Table.

(Fig 4b) showed the hub interaction of the pig*C* protein (red node) graphically with the top 11 nodes. The protein has 2721 edges, an expected 1126 edges, 199 nodes and an average of 27.3 nodes degree. The enrichment *p*-value for the predicted network is less than 1.0e-^16^, with an average local clustering coefficient of 0.701. These results showed that the protein is involved in various critical functions. Consequently, targeting such a protein can potentially cause the loss of function of the other associated proteins and thereby inhibit numerous biological pathways. Thus, it may be proposed as a potential therapeutic target.

#### 3.2.4. Shortlisting of a binding pocket and prediction of ligand

Furthermore, the DogSiteScorer was used to predict the protein’s binding pockets for the ligand for further protein structural characterization. Out of the 28 binding pockets identified, only one pocket was selected based on the highest average of simple and drug scores. The selected pocket was found with the highest drug score of 0.725 and a surface area of 1182.91 Å^2^. The descriptive details of the binding pocket can be found in S4 Fig. A potential ligand for the binding pocket was selected so that more detailed information on the protein’s structural properties could be obtained. For this purpose, MBC (4-Methoxy-2,2’-bipyrrole-5-carbaldehyde), MAP (2-Methyl-3-n-amyl-pyrrole) and ATP (Adenosine triphosphate) were used as ligands. These molecules are reported under R11662 in the KEGG database. MBC and MAP act as substrates and ATP as a cofactor for prodigiosin synthetase. The docking analysis revealed that all ligands bind with the proteins with favorable potencies. The ATP binds with the protein with a binding affinity of -4.64 kcal/mol, MBC with -6.04 kcal/mol and MAP with -4.83 kcal/mol, respectively.

**Fig 5:**
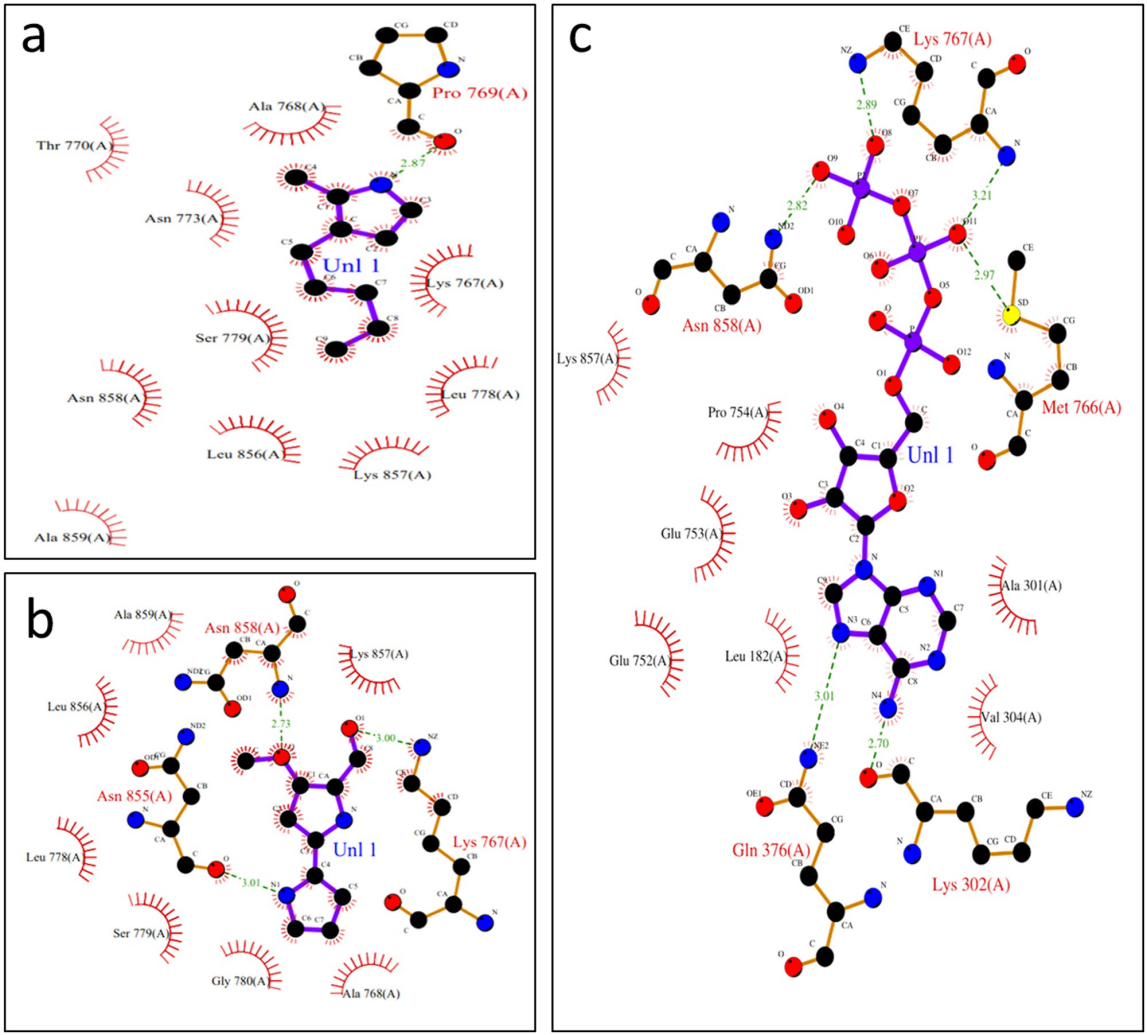
2D Intermolecular hydrogen bonding interactions of protein and ligand. Protein ligand interactions generated using LigPlot. **(a)** 2-Methyl-3-n-amyl-pyrrole (MAP), **(b)** 4-Methoxy-2,2’-bipyrrole-5-carbaldehyde (MBC), and **(c)** Adenosine triphosphate (ATP).

**Fig 6:**
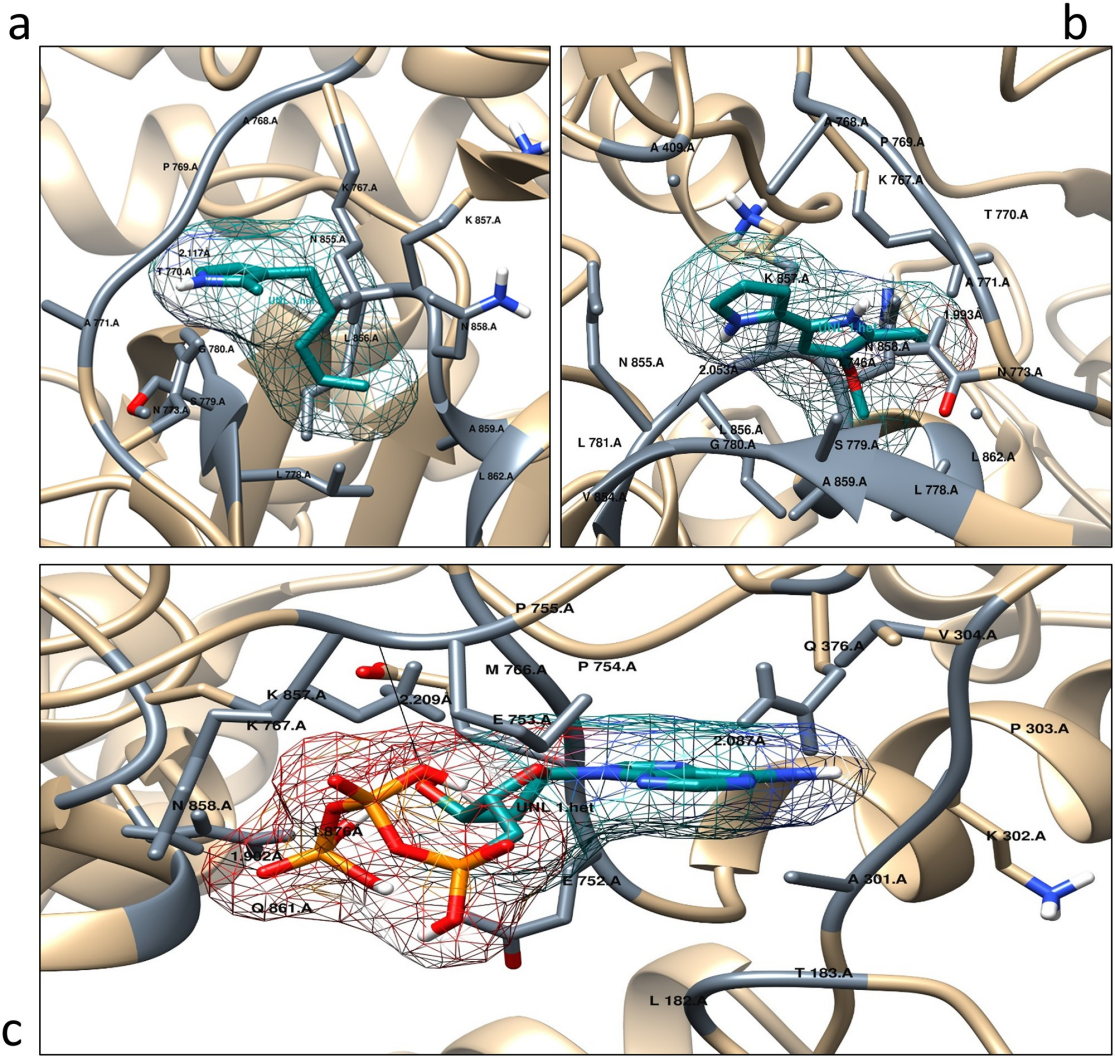
3D Intermolecular hydrogen bonding interactions of protein and ligand. Protein ligand interactions generated using LigPlot. **(a)** 2-Methyl-3-n-amyl-pyrrole (MAP), **(b)** 4-Methoxy-2,2’-bipyrrole-5-carbaldehyde (MBC), and **(c)** Adenosine triphosphate (ATP).

Additionally, inter-molecular hydrogen bonding interaction of the protein and the ligands are depicted. The protein-ligand interactions are visualized using Chimera and Ligplot. The 2D interactions of the Ligplot showed that the protein makes favorable hydrogen bonds with all three ligands as the bond lengths fall within 2.7 to 3.3 Å. The ATP forms the greatest number of hydrogen bonds with the amino acid residues of the protein, whereas MAP forms only a single hydrogen bond but numerous nonpolar interactions (Fig 5a). Furthermore, hydrogen bonds with amino acid residues of lysine and asparagine are common among MBC and ATP, Fig 5b and 5c respectively. The 3D interactions of the protein and the ligand were also generated using Chimera (Fig 6).

Table 6 shows the ligand’s intermolecular interactions with the protein’s binding site.

**Table 6:** Molecular Docking Analysis for reference compounds and substrates.

Compound	Ligand	Receptor	Distance (Å)	Binding Affinity (kcal/mol)
**MAP**	**Hydrogen Bond**			
	N	O-Pro 769	2.87	-4.83
	**Hydrophobic interactions**	Lys 767, Ala 768, Thr 770, Asn 773, Leu 778, Ser 779, Leu 856, Lys 857, Asn 858, Ala 859		
**MBC**	**Hydrogen Bond**			
	N2	O-Asn 855	3.01	-6.04
	O1	N- Asn 858	2.73	
	O2	N-Lys 767	3.00	
	**Hydrophobic interactions**	Ala 768, Leu 778, Ser 779, Gly 780, Leu 856, Lys 857, Ala 859		
**ATP**	**Hydrogen Bond**			
	O12	N-Lys 767	3.21	-4.64
	O9		2.89	
	O10	N-Asn 858	2.82	
	O12	S-Met 766	2.97	
	O4	O-Glu 753	2.54	
	N4	N-Gln376	3.01	
	N5	O-Lys 302	2.7	
	**Hydrophobic interactions**	Leu 182, Ala 301, Val 304, Glu 752, Pro 754, Lys 857		

#### 3.2.5. Virtual screening and ADMET profiling

**Fig 7:**
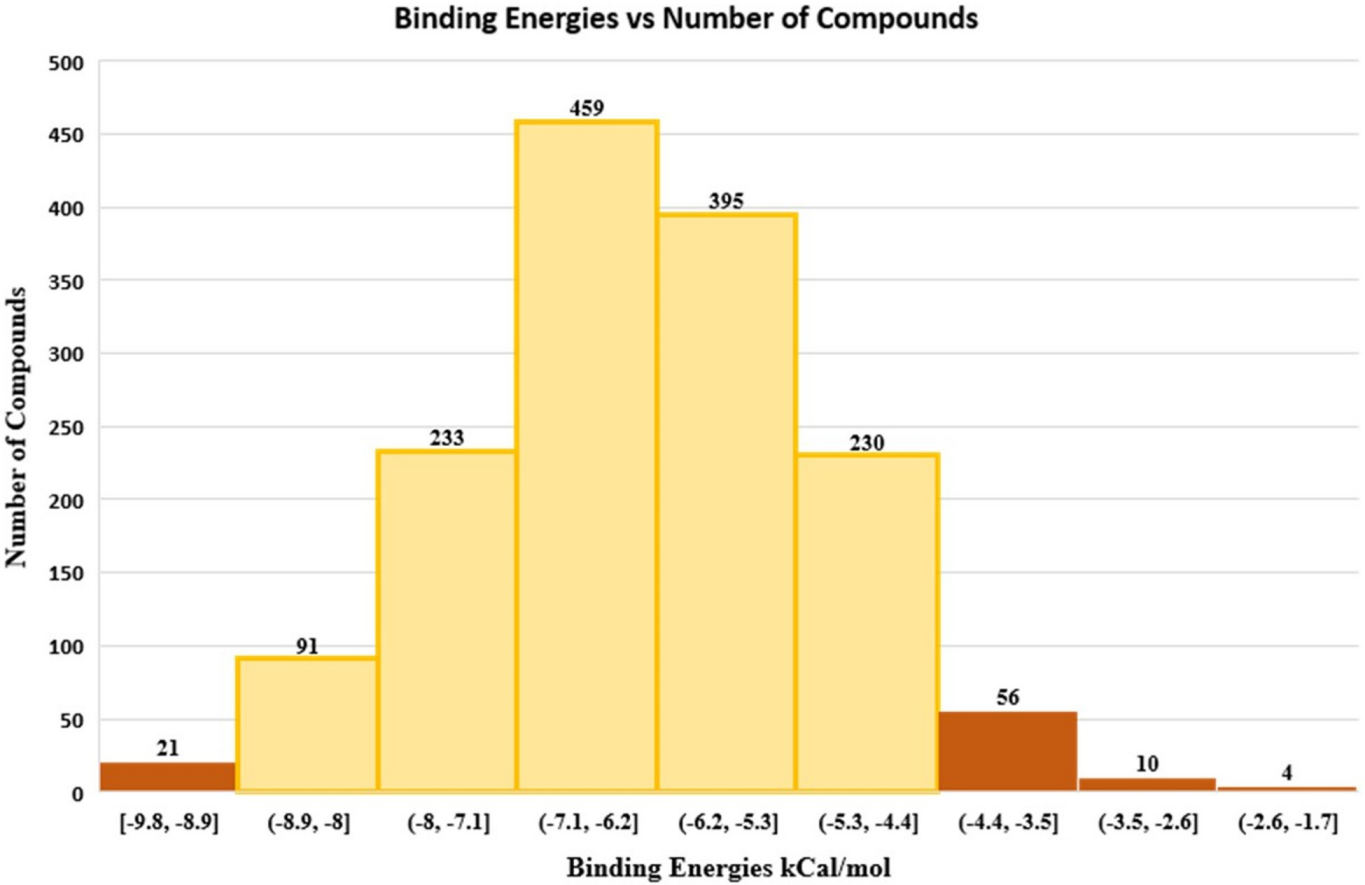
Virtual screening of ~1500 FDA approved drugs against prodigiosin synthetase. Results show that the most potential docked compounds within the range of -6.2 to -7.1 kcal/mol.

Virtual screening was performed to identify compounds with binding energies similar to the docked ligands which were used as a reference. Among 1500 Approved drug library, 854 compounds were clustered in two peaks with binding energies in the range of -5.3 to -7.1 kcal/mol (Fig 7).

Moreover, ADMET profiling was performed to shortlist compounds with potential binding affinity against Prodigiosin synthetase. Consequently, compounds DB00865, DB00821, DB00369, DB00423, DB00775 and DB00693 were shortlisted as favorable drug candidates Table 7. These six compounds were further shortlisted to two compounds which met certain selection criteria such as impermeable to the blood-brain barrier and high absorption through the GI tract i.e., DB00423 and DB00775. Compounds DB00423 and DB00775 showed estimated binding energies of -6.4 kcal/mol -6.7 kcal/mol, respectively. Additionally, both compounds showed no inhibition of the CYP1A2, CYP2C19, CYP2C9, CYP2D6 and CYP3A4 enzymes. The Swiss ADMET profiling showed that all three of these compounds follow the Lipinski rule and have estimated bioavailability scores of 0.55. These compounds showed favorable binding energies compared with the reference and therefore could serve as potent drug candidates against Prodigiosin synthetase.

**Table 7:** Properties of six shortlisted compounds from the approved library.

**Properties of Virtually Screened Compounds**						
**DrugBank ID**	DB00865	DB00821	DB00369	DB00423	DB00775	DB00693
**Generic Name**	Benzphetamine	Carprofen	Cidofovir	Merthocarbamol	Tirofiban	Fluorescein
**Pharmacokinetic Properties**						
**GI absorption**	High	High	Low	High	High	High
**BBB permeant**	Yes	Yes	No	No	No	Yes
**Drug—Likeness**						
**Lipinski**	Yes; 0 violation					
**Bioavailability Score**	0.55	0.85	0.11	0.55		
**Binding Affinity (kcal/mol)**	-5.0	-6.4	-5.4	-6.4	-6.7	-8.3
**Toxicity**						
**Ames Test**	Yes	No	No	Yes	No	No
**Hepatotoxic**	Yes	No	Yes	No	Yes	No
**Skin Sensitization**	Yes	No	No	No	No	Yes
**Max. tolerated dose (human) (mg/kg/day)**	0.609	0.765	1.341	1.223	0.173	0.279

#### 3.2.6. Conservancy analyses of predicted sequences with other strains

Furthermore, the results of the conservancy analysis showed that the pigC protein from the WW4 strain was locally aligned with the pigC protein of multiple different *Serratia marcescens* strains. It was observed that six proteins from different *S*. *marcescens* strains were aligned to the reference protein pigC protein. The proteins having sequence identity > 90% can be used for the pigC conservancy analysis, details of these proteins are provided in the S3 Table.

## 4. Discussion

*S*. *marcescens* is a Gram-negative *bacillus* and an opportunistic nosocomial pathogen causing infections of the urinary tract, blood, central nervous system and pneumonia [1]. *S*. *marcescens* harbor an inducible Amp*C* beta-lactamase present on the chromosome, which confers resistance to numerous antibiotics intrinsically [46]. Approximately, 200 *S*. *marcescens* outbreaks have been documented dating back to the 1950s [1]. As *S*. *marcescens* strains are multi-drug resistant therefore effective therapies such as novel medications and vaccines are needed.

Computational Biology is an emerging field of biological sciences that aids data analysis. New predictions are currently being made successfully using data mining techniques. The post-genomic era produced several genomic annotations that could not have been analyzed solely by humans. Computational Biology prioritizes potential drug targets to treat deadly diseases within the realm of infectious diseases. One such methodology is subtractive genomics and metabolic pathway analysis. The metabolic pathway analysis approach for the identification of novel drug targets is among the most cited strategies found in the literature. Environmental stress may cause upregulation of certain genes and the activation of selective metabolic pathways in bacteria. Such conditions may cause resistant pathogens to evolve with unique metabolic pathways in their dataset as shown in (Fig 2). Consequently, a metabolic pathway that has evolved for bacterial survival might be thought of as a possible therapeutic target. Some bacterial strains may be more sensitive to drugs if they lack a particular metabolic pathway in their genome. Significantly, subtractive genomics is widely used for the prioritization of potential drug targets against various hazardous pathogens [47, 48].

Therefore, in the current study, a comparative metabolic pathway analysis along with subtractive genomics was applied to identify unique proteins that may serve as potential drug targets against *S*. *marcescens*. Using this technique, the pathogen’s unique metabolic pathways were identified, and essential proteins were prioritized as potential drug targets. Following this strategy, pathway 00333 (Prodigiosin biosynthesis) was found unique to a single strain (i.e., WW4) as shown in (Fig 2). We propose that inhibiting the Prodigiosin biosynthesis pathway by targeting any of the pathway’s essential proteins may result in a potential treatment against this particular strain of *S*. *marcescens*. The druggability analysis retrieved ‘no hits’ from the database meaning that the Prodigiosin synthetase protein can serve as a novel drug target [49]. Furthermore, DEG shortlisted Prodigiosin synthetase as an essential protein associated with the prodigiosin biosynthesis pathway out of the six proteins as shown in the Table 3. Prodigiosin synthetase is involved in the final condensation step that results in the production of the antimicrobials. As the pathogen invests a huge amount of energy in the biosynthesis of Prodigiosin, therefore it can be inferred that it is critical for the survival of *S*. *marcescens*, particularly during interspecies competition. Targeting such a protein might inhibit the pathway thus turning the bacteria susceptible to the treatment (i.e. drug).

Various bacteria including *S*. *marcescens* and those belonging to the *Streptomycetaceae* and *Pseudoalteromonadaceae* families produce Prodiginines [50]. The awareness of the benefits of this alkaloid has led to the research on its production at the industrial level. The ongoing research showed that fatty acids from powdered peanut broth nurtured the growth of *S*. *marcescens* and elevated Prodigiosin production [51].

Thus, these studies concentrated on the benefits of this alkaloid for humans. However, it is also important to understand the threats associated with a *S*. *marcescens* infection. For example, this bacterium may pose to our body’s natural flora as they are antimicrobial in nature. Knowledge of prodigiosin synthetase that synthesizes prodigiosin may help alleviate prevalence of infections by *Serratia marcescens*. A study has revealed that mutagenesis, particularly a 17 bp deletion in the phosphoenolpyruvate (PEP) domain of the protein resulted in the truncation and loss of function of the domain. As a result, the bacteria (i.e. *S*. *marcescens*) not only failed to synthesize but also failed to secrete Prodigiosin since the macrovesicle formation was inhibited [52]. This showed that this protein may serve as a viable drug target as it is quintessential in the synthesis of Prodigiosin.

Additionally, de novo modeling was used to model the structure of Prodigiosin synthetase as shown (Fig 3a). The structure was validated using Robetta server (Fig 3b), Verify 3D (Fig 3c), PSIPRED as shown in S1 Fig and Procheck. S2 Fig shows that the Ramachandran plot generated using Procheck classified 91.5% of the amino acid residues of the protein in the core-allowed region. Similar results were obtained in a previous study [53]. The Verify 3D assigned a quality score of 95.83% to the model as shown in (Fig 3c). The model is considered reliable when the compatibility scores are higher than 80%. This further confirmed that the generated model is accurate [34]. The prediction of the binding site and ligands provided detailed information regarding the protein structure. PSORTb analysis showed the suitability of the protein as a drug target and it was concluded that it is cytoplasmic (Fig 4a). The physicochemical analysis showed that the protein is unstable. It is in-line with the previous studies that hub proteins are more disordered than end proteins [54]. Such proteins are suitable targets for inhibition as they have been associated with signaling, regulatory pathways and cancer [55]. The targeted hub protein was shown to interact with 198 proteins through network topology analysis as shown in (Fig 4b). The protein has an aliphatic index of 88.41 and this means that the protein is thermally stable.

Significantly, the docking studies showed that ATP binds with the protein with a binding affinity of -4.64 kcal/mol, MBC with -6.04 kcal/mol and MAP with -4.83 kcal/mol. The same parameters that were used for the docking of these ligands were used for the virtual screening of ~1500 approved compounds library. About 854 compounds out of 1500 were found with binding energies in the range of -5.3 to -7.1kcal/mol. Compounds shortlisted for further analysis are highlighted as yellow bars in (Fig 7). A total of six compounds were shortlisted with binding energies in the range of -5.0 to -8.3 kcal/mol i.e., compounds DB00865, DB00821, DB00369, DB00423, DB00775 and DB00693. These six compounds were further shortlisted to two compounds according to certain criteria such as impermeable to the blood-brain barrier and high absorption through the GI tract i.e., DB00423 and DB00775 as shown in Table 7. As they have favorable binding energies they may serve as good drug candidates against Prodigiosin synthetase.

The results of the conservancy analysis part of the study showed that Prodigiosin Synthetase (PigC) displayed high conservancy pattern with homologues of *Serratia marcescens* strains from different geographical locations. The predicted protein should be conserved among the bacterial strains of specific pathogen to ensure that it can serve as an effective drug or vaccine target across multiple strains [56]. Hence, PigC protein might be a potent broad-spectrum drug target against *Serratia marcescens*.

Nevertheless, the study has some limitations as all methods were carried out on a computational approach. Further complementary experimental studies are recommended to validate these findings. *In vitro* and *in vivo* studies are the suggested follow-up for future collaborative research among the scientific community.

## 5. Conclusion

Pathogenic genome and proteome analysis helped in drug target identification. In this research, a subtractive genomic and metabolic pathway analysis technique was performed for the prediction of a non-homologous, essential, druggable protein against *S*. *marcescens*. Consequently, the protein Prodigiosin synthetase was proposed as a potential drug target. This protein is involved in a network with 198 other proteins, therefore targeting it may help eradicate the pathogen from the respective host. Moreover, using the molecular docking approach for the identified drug target, one can identify and select molecules such as DB00423 and DB00775 as the ones with the most favorable binding affinities with the target active site residues. This study may enable future researchers to produce efficacious drugs and vaccines against strain-specific *S*. *marcescens*.

## Supporting information

S1 Appendix Script for subtractive genomics. (DOCX)

S1 Fig PsiPred results of protein. (TIF)

S2 Fig ProCheck results of protein. (TIF)

S3 Fig InterProScan results. (A) The image shows the accession numbers of the functional domains while (B) image tabulates the names of the domains.
(TIF)

S4 Fig DogSiteScorer results. The ligand binding pocket in protein predicted through DoGSite Scorer. Detailed description related to binding pocket and amino acids found in active are mentioned.
(TIF)

S1 Table KEGG pathway IDs displayed in the heatmap. (DOCX)

S2 Table STRING interaction network. Table showing the detailed annotation of the direct interaction formed by the hub protein (PigC).
(DOCX)

S3 Table The table shows the conservancy analysis results of the prodigiosin synthetase (PigC) protein in other *Serratia marcescens* strains. (DOCX)

S1 Graphical abstract (TIF)
