# Supplementary material for: Proteogenomic analysis of Serratia marcescens using computational subtractive genomics approach
Source: PLoS One. 2023 Apr 10;18(4):e0283993. doi: 10.1371/journal.pone.0283993 (PMC10085029; doi:10.1371/journal.pone.0283993)
Supplement: S1 Appendix — (DOCX) [file pone.0283993.s001.docx]

**Commands for Subtractive Genomics**

1. Filter Pathway data from htext file

Grep “PATH:” file.keg > Pathwaysmar

Grep “PATH:” file.keg > Pathwaysmac

Grep “PATH:” file.keg > Pathwaysmw

1. Use Block Selection mode in kate to retrieve only ids of pathways
2. Use Diffuse tool to find unique/missing pathways from files created in step 2

Diffuse file1 file2 file3

1. Obtain sequences of unique /missing pathway proteins using KEGG
2. Create database containing human proteome sequences

makeblastdb -in GRCh38_latest_protein.faa -dbtype prot

1. Blast file from step 4 against this database to find non-homologous proteins

blastp -query (name of file from step 4) -db GRCh38_latest_protein.faa -out (name of output file) -evalue 1e-3

1. Create database containing DEG protein sequences

makeblastdb -in DEG10.aa -dbtype prot

1. Blast output file from step 6 against database in step 7 to find essential proteins

blastp -query (output file from step 6) -db DEG10.aa -out (name of output file) -evalue 1e-5

1. Create database containing DrugBank Database (All Targets)

makeblastdb -in all_target.fasta -dbtype prot

1. Blast output file from step 8 against database in step 9 to find essential proteins

blastp -query (output file from step 8) -db all_target.fasta -out (name of output file) -evalue 1e-5
