# Supplementary figures and images for: Proteogenomic analysis of Serratia marcescens using computational subtractive genomics approach
Source: PLoS One. 2023 Apr 10;18(4):e0283993. doi: 10.1371/journal.pone.0283993 (PMC10085029; doi:10.1371/journal.pone.0283993)

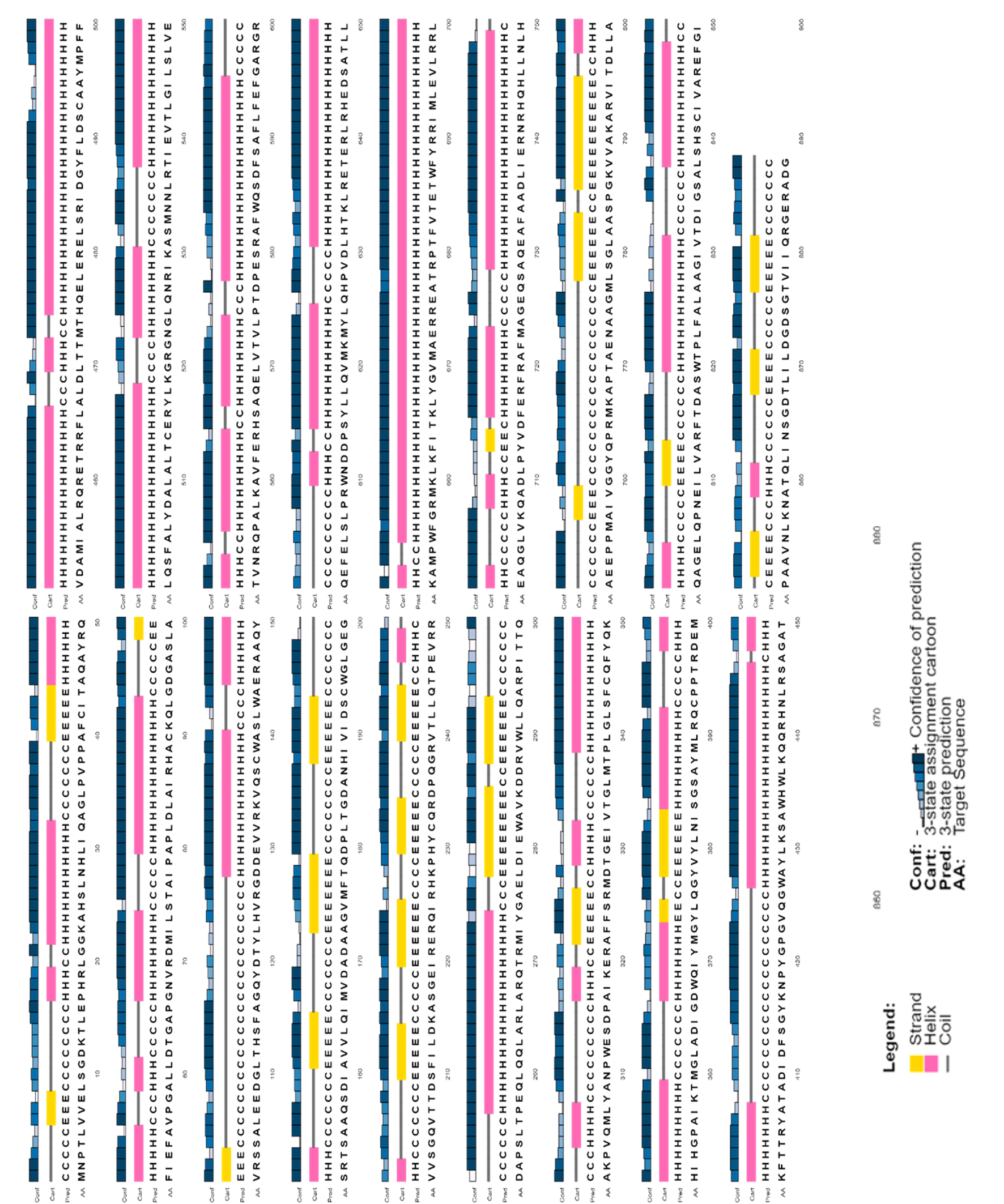

Supplement: S1 Fig — (TIF) [file pone.0283993.s002.tif]

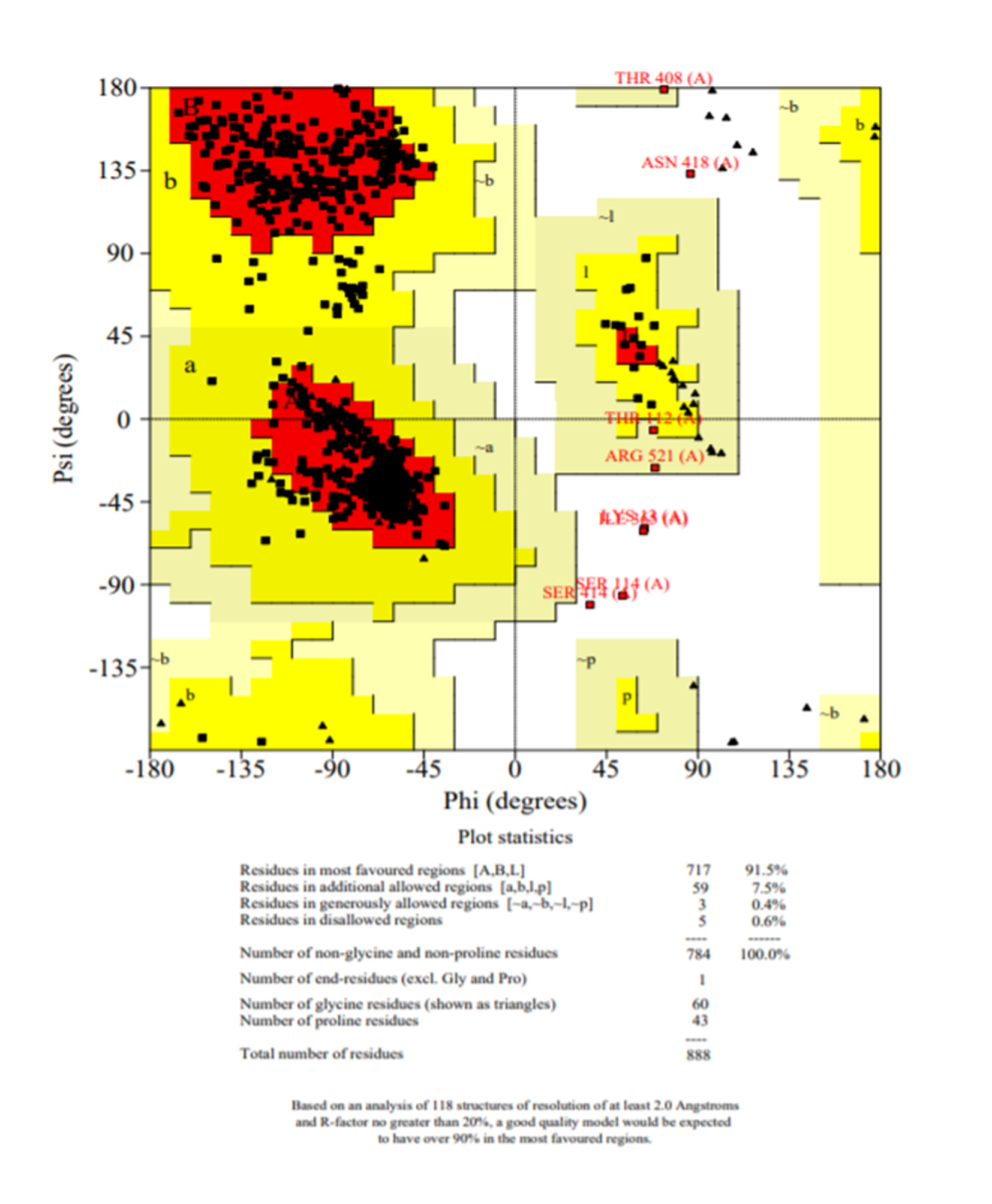

Supplement: S2 Fig — (TIF) [file pone.0283993.s003.tif]

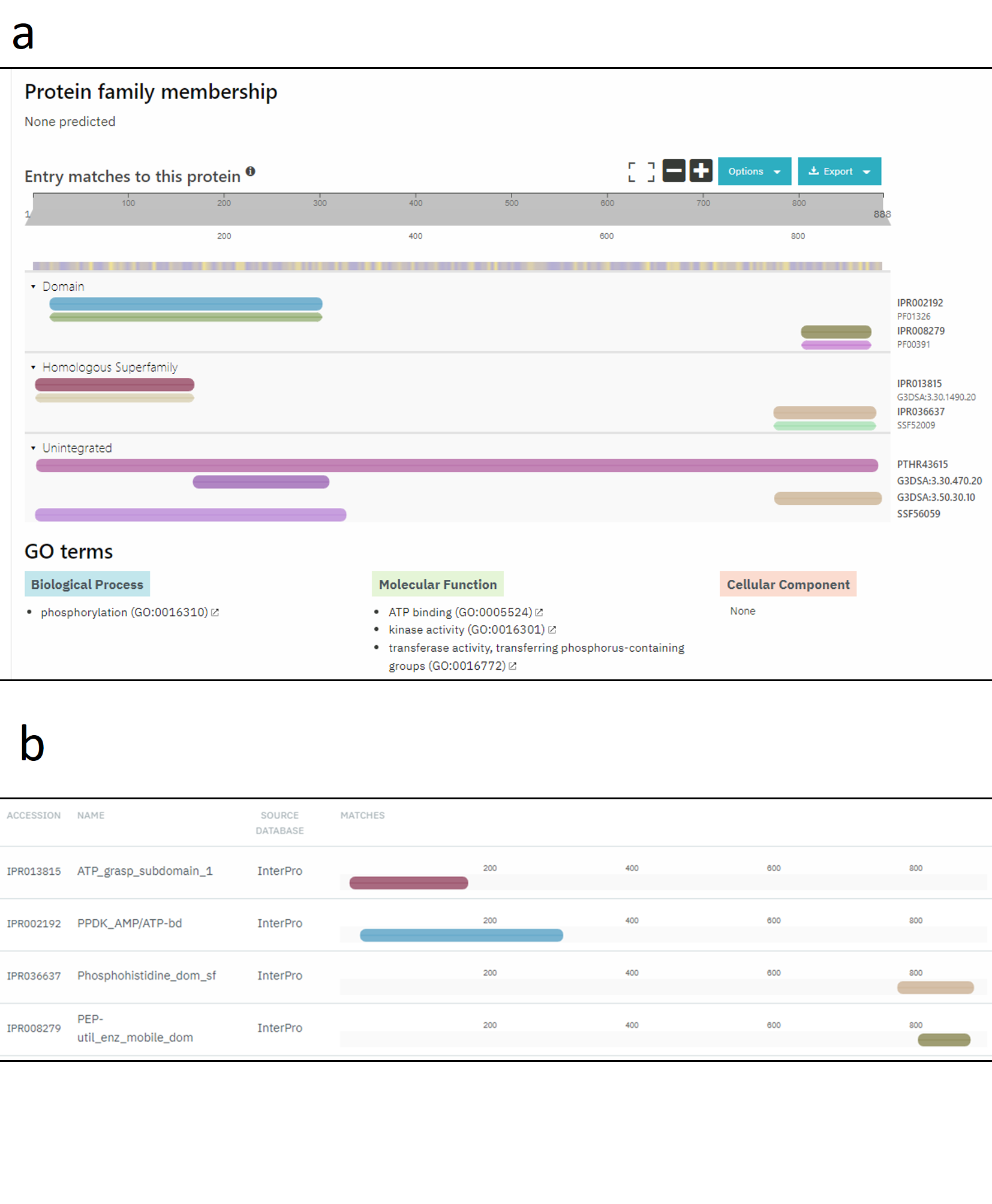

Supplement: S3 Fig — (A) The image shows the accession numbers of the functional domains while (B) image tabulates the names of the domains. (TIF) [file pone.0283993.s004.tif]

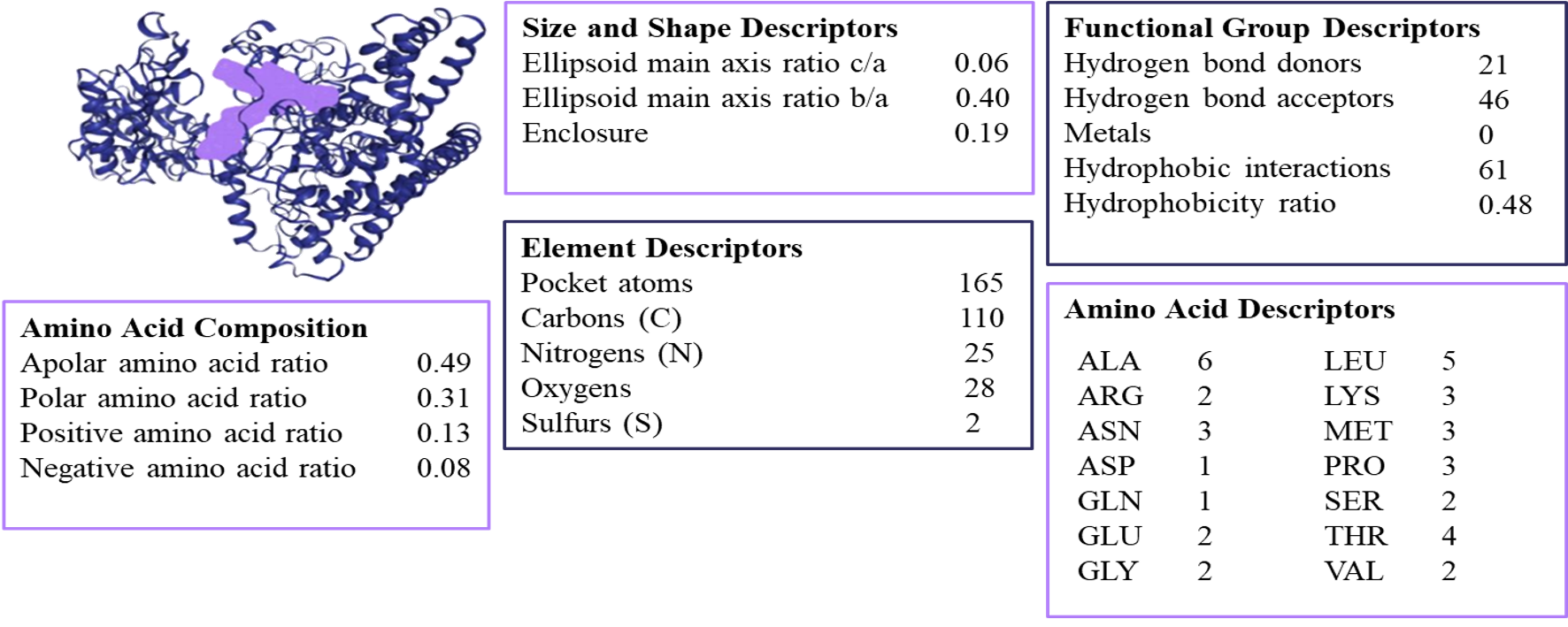

Supplement: S4 Fig — The ligand binding pocket in protein predicted through DoGSite Scorer. Detailed description related to binding pocket and amino acids found in active are mentioned. (TIF) [file pone.0283993.s005.tif]

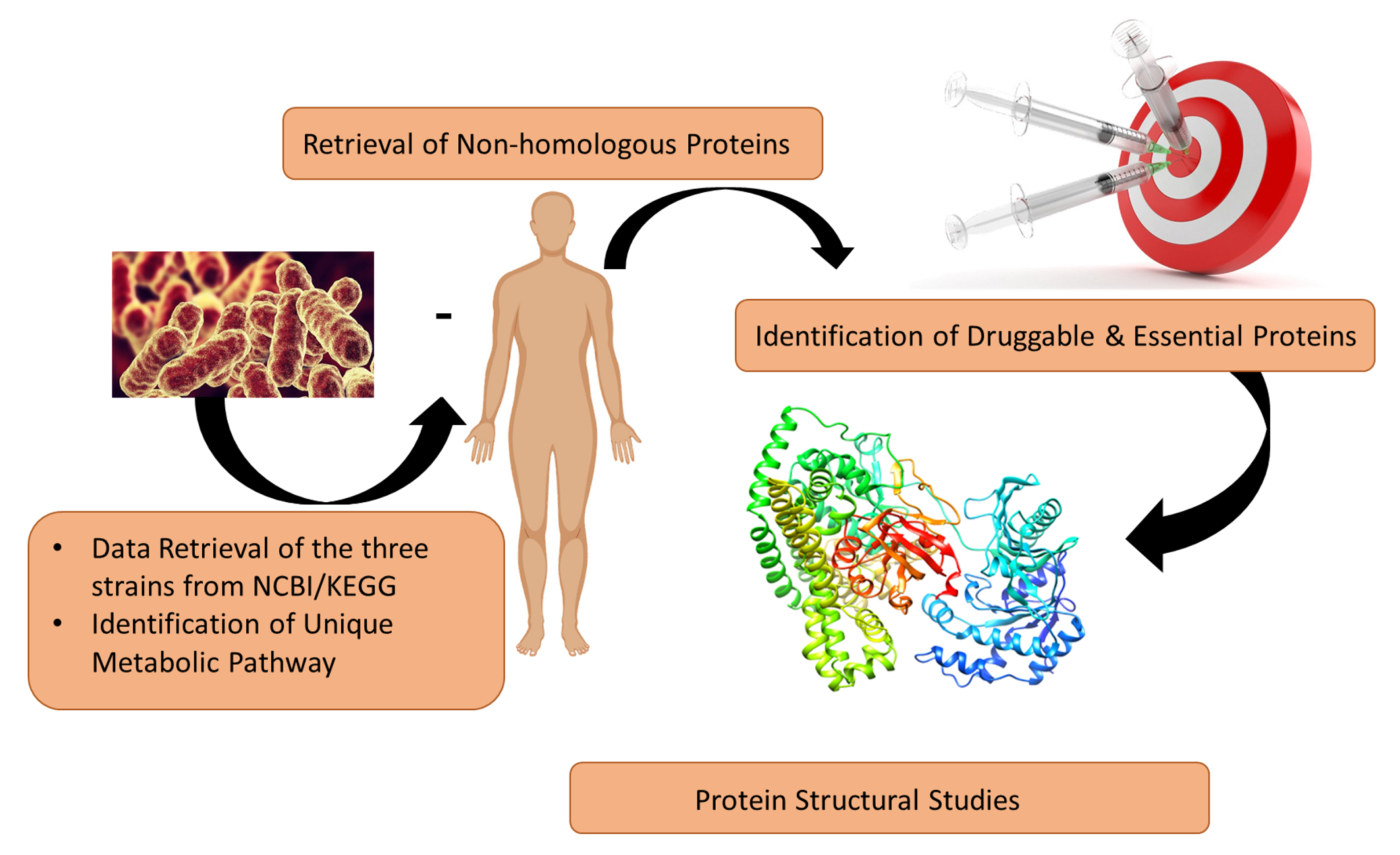

Supplement: S1 Graphical abstract — (TIF) [file pone.0283993.s009.tif]
