# Supplementary material for: Proteogenomic analysis of Serratia marcescens using computational subtractive genomics approach
Source: PLoS One. 2023 Apr 10;18(4):e0283993. doi: 10.1371/journal.pone.0283993 (PMC10085029; doi:10.1371/journal.pone.0283993)
Supplement: S1 Table — (DOCX) [file pone.0283993.s006.docx]

| **ID Name of Pathway** |
| --- |
| 00643 Styrene degradation |
| 00624 Polycyclic aromatic hydrocarbon degradation |
| 00333 Prodigiosin Biosynthesis |
| 01503 Cationic antimicrobial peptide resistance |
| 01502 Vancomycin resistance |
| 01501 Beta-Lactam resistance |
| 02040 Flagellar assembly |
| 02030 Bacterial chemotaxis |
| 02024 Quorum sensing |
| 02020 Two-component system |
| 03070 Bacterial secretion system |
| 02060 Phosphotransferase system |
| 02010 ABC transporters |
| 03440 Homologous recombination |
| 03430 Mismatch repair |
| 03420 Nucleotide excision repair |
| 03410 Base excision repair |
| 03030 DNA replication |
| 03018 RNA degradation |
| 04122 Sulfur relay system |
| 03060 Protein export |
| 00970 Aminoacyl-tRNA biosynthesis |
| 03010 Ribosome |
| 03020 RNA polymerase |
| 00626 Naphthalene degradation |
| 00930 Caprolactam degradation |
| 00791 Atrazine degradation |
| 00633 Nitrotoluene degradation |
| 00622 Xylene degradation |
| 00623 Toluene degradation |
| 00361 Chlorocyclohexane & chlorobenzene degradation |
| 00625 Chloroalkane & chloroalkene degradation |
| 00364 Fluorobenzoate degradation |
| 00627 Aminobenzoate degradation |
| 00362 Benzoate degradation |
| 00997 Biosynthesis of various secondary metabolites |
| 00401 Novobiocin biosynthesis |
| 00525 Acarbose and validamycin biosynthesis |
| 00521 Streptomycin biosynthesis |
| 00261 Monobactam biosynthesis |
| 00332 Carbapenem biosynthesis |
| 01053 Biosynthesis of siderophore group nonribosomal peptides |
| 00523 Polyketide sugar unit biosynthesis |
| 00281 Geraniol degradation |
| 00903 Limonene and pinene degradation |
| 00900 Terpenoid backbone biosynthesis |
| 00130 Ubiquinone /terpenoid-quinone biosynthesis |
| 00860 Porphyrin and chlorophyll metabolism |
| 00670 One carbon pool by folate |
| 00790 Folate biosynthesis |
| 00785 Lipoic acid metabolism |
| 00780 Biotin metabolism |
| 00770 Pantothenate and CoA biosynthesis |
| 00760 Nicotinate and nicotinamide metabolism |
| 00750 Vitamin B6 metabolism |
| 00740 Riboflavin metabolism |
| 00730 Thiamine metabolism |
| 00511 Other glycan degradation |
| 00550 Peptidoglycan biosynthesis |
| 00541 O-Antigen nucleotide sugar biosynthesis |
| 00542 O-Antigen repeat unit biosynthesis |
| 00540 Lipopolysaccharide biosynthesis |
| 00480 Glutathione metabolism |
| 00470 D-Amino acid metabolism |
| 00460 Cyanoamino acid metabolism |
| 00450 Selenocompound metabolism |
| 00440 Phosphonate and phosphinate metabolism |
| 00430 Taurine and hypotaurine metabolism |
| 00410 beta-Alanine metabolism |
| 00400 Phenylalanine, tyrosine & tryptophan biosynthesis |
| 00380 Tryptophan metabolism |
| 00360 Phenylalanine metabolism |
| 00350 Tyrosine metabolism |
| 00340 Histidine metabolism |
| 00330 Arginine and proline metabolism |
| 00220 Arginine biosynthesis |
| 00310 Lysine degradation |
| 00300 Lysine biosynthesis |
| 00290 Valine, leucine and isoleucine biosynthesis |
| 00280 Valine, leucine and isoleucine degradation |
| 00270 Cysteine and methionine metabolism |
| 00260 Glycine, serine and threonine metabolism 00250 Alanine, aspartate and glutamate metabolism |
| 00240 Pyrimidine metabolism |
| 00230 Purine metabolism |
| 01040 Biosynthesis of unsaturated fatty acids |
| 00592 alpha-Linolenic acid metabolism |
| 00591 Linoleic acid metabolism |
| 00590 Arachidonic acid metabolism |
| 00565 Ether lipid metabolism |
| 00564 Glycerophospholipid metabolism |
| 00561 Glycerolipid metabolism |
| 00071 Fatty acid degradation |
| 00061 Fatty acid biosynthesis |
| 00920 Sulfur metabolism |
| 00910 Nitrogen metabolism |
| 00680 Methane metabolism |
| 00190 Oxidative phosphorylation |
| 00562 Inositol phosphate metabolism |
| 00660 C5-Branched dibasic acid metabolism |
| 00650 Butanoate metabolism |
| 00640 Propanoate metabolism |
| 00630 Glyoxylate and dicarboxylate metabolism |
| 00620 Pyruvate metabolism |
| 00520 Amino sugar and nucleotide sugar metabolism |
| 00500 Starch and sucrose metabolism |
| 00053 Acsorbate and aldarate metabolism |
| 00052 Galactose metabolism |
| 00051 Fructose and mannose metabolism |
| 00040 Pentose and glucuronate interconversions |
| 00030 Pentose phosphate pathway |
| 00010 Glycolysis / Gluconeogenesis |
| 00020 Citrate Cycle |
