# Supplementary material for: Proteogenomic analysis of Serratia marcescens using computational subtractive genomics approach
Source: PLoS One. 2023 Apr 10;18(4):e0283993. doi: 10.1371/journal.pone.0283993 (PMC10085029; doi:10.1371/journal.pone.0283993)
Supplement: S2 Table — Table showing the detailed annotation of the direct interaction formed by the hub protein (PigC). (DOCX) [file pone.0283993.s007.docx]

| node1 | node2 | node1 annotation | node2 annotation | score |
| --- | --- | --- | --- | --- |
| pigC | ESN61248.1 | Pyruvate, water dikinase, phosphoenolpyruvate--protein phosphotransferase; Involved in the biosynthesis of 2-methyl-3-n-amyl-pyrrole (MAP), one of the terminal products involved in the biosynthesis of the red antibiotic prodigiosin (Pig). Catalyzes the transfer of 2-methyl-3- n-amyl-pyrrole (MAP) to 4-methoxy-2,2'-bipyrrole-5-carbaldehyde (MBC) to yield prodigiosin | Phosphopyruvate hydratase; Catalyzes the reversible conversion of 2-phosphoglycerate into phosphoenolpyruvate. It is essential for the degradation of carbohydrates via glycolysis | 0.983 |
| pigC | ESN61628.1 | Pyruvate, water dikinase, phosphoenolpyruvate--protein phosphotransferase; Involved in the biosynthesis of 2-methyl-3-n-amyl-pyrrole (MAP), one of the terminal products involved in the biosynthesis of the red antibiotic prodigiosin (Pig). Catalyzes the transfer of 2-methyl-3- n-amyl-pyrrole (MAP) to 4-methoxy-2,2'-bipyrrole-5-carbaldehyde (MBC) to yield prodigiosin | annotation not available | 0.978 |
| pigC | ESN62118.1 | Pyruvate, water dikinase, phosphoenolpyruvate--protein phosphotransferase; Involved in the biosynthesis of 2-methyl-3-n-amyl-pyrrole (MAP), one of the terminal products involved in the biosynthesis of the red antibiotic prodigiosin (Pig). Catalyzes the transfer of 2-methyl-3- n-amyl-pyrrole (MAP) to 4-methoxy-2,2'-bipyrrole-5-carbaldehyde (MBC) to yield prodigiosin | Belongs to the pyruvate kinase family | 0.990 |
| pigC | ESN63896.1 | Pyruvate, water dikinase, phosphoenolpyruvate--protein phosphotransferase; Involved in the biosynthesis of 2-methyl-3-n-amyl-pyrrole (MAP), one of the terminal products involved in the biosynthesis of the red antibiotic prodigiosin (Pig). Catalyzes the transfer of 2-methyl-3- n-amyl-pyrrole (MAP) to 4-methoxy-2,2'-bipyrrole-5-carbaldehyde (MBC) to yield prodigiosin | Hypothetical protein; Uncharacterized protein; KEGG: malic enzyme | 0.995 |
| pigC | eno | Pyruvate, water dikinase, phosphoenolpyruvate--protein phosphotransferase; Involved in the biosynthesis of 2-methyl-3-n-amyl-pyrrole (MAP), one of the terminal products involved in the biosynthesis of the red antibiotic prodigiosin (Pig). Catalyzes the transfer of 2-methyl-3- n-amyl-pyrrole (MAP) to 4-methoxy-2,2'-bipyrrole-5-carbaldehyde (MBC) to yield prodigiosin | Phosphopyruvate hydratase; Catalyzes the reversible conversion of 2-phosphoglycerate into phosphoenolpyruvate. It is essential for the degradation of carbohydrates via glycolysis | 0.983 |
| pigC | pyk | Pyruvate, water dikinase, phosphoenolpyruvate--protein phosphotransferase; Involved in the biosynthesis of 2-methyl-3-n-amyl-pyrrole (MAP), one of the terminal products involved in the biosynthesis of the red antibiotic prodigiosin (Pig). Catalyzes the transfer of 2-methyl-3- n-amyl-pyrrole (MAP) to 4-methoxy-2,2'-bipyrrole-5-carbaldehyde (MBC) to yield prodigiosin | Belongs to the pyruvate kinase family | 0.990 |
| pigC | ESN64983.1 | Pyruvate, water dikinase, phosphoenolpyruvate--protein phosphotransferase; Involved in the biosynthesis of 2-methyl-3-n-amyl-pyrrole (MAP), one of the terminal products involved in the biosynthesis of the red antibiotic prodigiosin (Pig). Catalyzes the transfer of 2-methyl-3- n-amyl-pyrrole (MAP) to 4-methoxy-2,2'-bipyrrole-5-carbaldehyde (MBC) to yield prodigiosin | annotation not available | 0.981 |
| pigC | nifJ | Pyruvate, water dikinase, phosphoenolpyruvate--protein phosphotransferase; Involved in the biosynthesis of 2-methyl-3-n-amyl-pyrrole (MAP), one of the terminal products involved in the biosynthesis of the red antibiotic prodigiosin (Pig). Catalyzes the transfer of 2-methyl-3- n-amyl-pyrrole (MAP) to 4-methoxy-2,2'-bipyrrole-5-carbaldehyde (MBC) to yield prodigiosin | annotation not available | 0.981 |
| pigC | maeA | Pyruvate, water dikinase, phosphoenolpyruvate--protein phosphotransferase; Involved in the biosynthesis of 2-methyl-3-n-amyl-pyrrole (MAP), one of the terminal products involved in the biosynthesis of the red antibiotic prodigiosin (Pig). Catalyzes the transfer of 2-methyl-3- n-amyl-pyrrole (MAP) to 4-methoxy-2,2'-bipyrrole-5-carbaldehyde (MBC) to yield prodigiosin | Malate dehydrogenase (oxaloacetate-decarboxylating); NAD-dependent malic enzyme; KEGG: malate dehydrogenase | 0.989 |
| pigC | pigB | Pyruvate, water dikinase, phosphoenolpyruvate--protein phosphotransferase; Involved in the biosynthesis of 2-methyl-3-n-amyl-pyrrole (MAP), one of the terminal products involved in the biosynthesis of the red antibiotic prodigiosin (Pig). Catalyzes the transfer of 2-methyl-3- n-amyl-pyrrole (MAP) to 4-methoxy-2,2'-bipyrrole-5-carbaldehyde (MBC) to yield prodigiosin | Hypothetical protein; Involved in the biosynthesis of 2-methyl-3-n-amyl-pyrrole (MAP), one of the terminal products involved in the biosynthesis of the red antibiotic prodigiosin (Pig). Catalyzes the oxidation of dihydro form of MAP (H2MAP) to yield MAP | 0.970 |

| node1 | node2 | node1 annotation | node2 annotation | score |
| --- | --- | --- | --- | --- |
| pigC | ESN62118.1 | Pyruvate, water dikinase, phosphoenolpyruvate--protein phosphotransferase; Involved in the biosynthesis of 2-methyl-3-n-amyl-pyrrole (MAP), one of the terminal products involved in the biosynthesis of the red antibiotic prodigiosin (Pig). Catalyzes the transfer of 2-methyl-3- n-amyl-pyrrole (MAP) to 4-methoxy-2,2'-bipyrrole-5-carbaldehyde (MBC) to yield prodigiosin | Belongs to the pyruvate kinase family | 0.990 |
| pigC | ESN63896.1 | Pyruvate, water dikinase, phosphoenolpyruvate--protein phosphotransferase; Involved in the biosynthesis of 2-methyl-3-n-amyl-pyrrole (MAP), one of the terminal products involved in the biosynthesis of the red antibiotic prodigiosin (Pig). Catalyzes the transfer of 2-methyl-3- n-amyl-pyrrole (MAP) to 4-methoxy-2,2'-bipyrrole-5-carbaldehyde (MBC) to yield prodigiosin | Hypothetical protein; Uncharacterized protein; KEGG: malic enzyme | 0.995 |

| node1 | node2 | node1 annotation | node2 annotation | score |
| --- | --- | --- | --- | --- |
| pigC | ESN63896.1 | Pyruvate, water dikinase, phosphoenolpyruvate--protein phosphotransferase; Involved in the biosynthesis of 2-methyl-3-n-amyl-pyrrole (MAP), one of the terminal products involved in the biosynthesis of the red antibiotic prodigiosin (Pig). Catalyzes the transfer of 2-methyl-3- n-amyl-pyrrole (MAP) to 4-methoxy-2,2'-bipyrrole-5-carbaldehyde (MBC) to yield prodigiosin | Hypothetical protein; Uncharacterized protein; KEGG: malic enzyme | 0.995 |

| node1 | node2 | node1 annotation | node2 annotation | score |
| --- | --- | --- | --- | --- |
| pigC | ESN64464.1 | Pyruvate, water dikinase, phosphoenolpyruvate--protein phosphotransferase; Involved in the biosynthesis of 2-methyl-3-n-amyl-pyrrole (MAP), one of the terminal products involved in the biosynthesis of the red antibiotic prodigiosin (Pig). Catalyzes the transfer of 2-methyl-3- n-amyl-pyrrole (MAP) to 4-methoxy-2,2'-bipyrrole-5-carbaldehyde (MBC) to yield prodigiosin | Dihydrolipoyllysine-residue succinyltransferase; E2 component of the 2-oxoglutarate dehydrogenase (OGDH) complex which catalyzes the second step in the conversion of 2- oxoglutarate to succinyl-CoA and CO(2) | 0.619 |
| pigC | ESN64479.1 | Pyruvate, water dikinase, phosphoenolpyruvate--protein phosphotransferase; Involved in the biosynthesis of 2-methyl-3-n-amyl-pyrrole (MAP), one of the terminal products involved in the biosynthesis of the red antibiotic prodigiosin (Pig). Catalyzes the transfer of 2-methyl-3- n-amyl-pyrrole (MAP) to 4-methoxy-2,2'-bipyrrole-5-carbaldehyde (MBC) to yield prodigiosin | Betaine-aldehyde dehydrogenase; Belongs to the aldehyde dehydrogenase family | 0.627 |
| pigC | ESN64512.1 | Pyruvate, water dikinase, phosphoenolpyruvate--protein phosphotransferase; Involved in the biosynthesis of 2-methyl-3-n-amyl-pyrrole (MAP), one of the terminal products involved in the biosynthesis of the red antibiotic prodigiosin (Pig). Catalyzes the transfer of 2-methyl-3- n-amyl-pyrrole (MAP) to 4-methoxy-2,2'-bipyrrole-5-carbaldehyde (MBC) to yield prodigiosin | Phosphogluconate dehydrogenase (decarboxylating); Catalyzes the oxidative decarboxylation of 6-phosphogluconate to ribulose 5-phosphate and CO(2), with concomitant reduction of NADP to NADPH | 0.512 |
| pigC | ESN64529.1 | Pyruvate, water dikinase, phosphoenolpyruvate--protein phosphotransferase; Involved in the biosynthesis of 2-methyl-3-n-amyl-pyrrole (MAP), one of the terminal products involved in the biosynthesis of the red antibiotic prodigiosin (Pig). Catalyzes the transfer of 2-methyl-3- n-amyl-pyrrole (MAP) to 4-methoxy-2,2'-bipyrrole-5-carbaldehyde (MBC) to yield prodigiosin | Hypothetical protein; Uncharacterized protein; KEGG: thioesterase | 0.555 |
| pigC | ESN64534.1 | Pyruvate, water dikinase, phosphoenolpyruvate--protein phosphotransferase; Involved in the biosynthesis of 2-methyl-3-n-amyl-pyrrole (MAP), one of the terminal products involved in the biosynthesis of the red antibiotic prodigiosin (Pig). Catalyzes the transfer of 2-methyl-3- n-amyl-pyrrole (MAP) to 4-methoxy-2,2'-bipyrrole-5-carbaldehyde (MBC) to yield prodigiosin | Phenylalanine racemase (atp-hydrolyzing); Belongs to the ATP-dependent AMP-binding enzyme family | 0.445 |
| pigC | ESN64547.1 | Pyruvate, water dikinase, phosphoenolpyruvate--protein phosphotransferase; Involved in the biosynthesis of 2-methyl-3-n-amyl-pyrrole (MAP), one of the terminal products involved in the biosynthesis of the red antibiotic prodigiosin (Pig). Catalyzes the transfer of 2-methyl-3- n-amyl-pyrrole (MAP) to 4-methoxy-2,2'-bipyrrole-5-carbaldehyde (MBC) to yield prodigiosin | Acetoin dehydrogenase; Belongs to the short-chain dehydrogenases/reductases (SDR) family | 0.421 |
| pigC | ESN64553.1 | Pyruvate, water dikinase, phosphoenolpyruvate--protein phosphotransferase; Involved in the biosynthesis of 2-methyl-3-n-amyl-pyrrole (MAP), one of the terminal products involved in the biosynthesis of the red antibiotic prodigiosin (Pig). Catalyzes the transfer of 2-methyl-3- n-amyl-pyrrole (MAP) to 4-methoxy-2,2'-bipyrrole-5-carbaldehyde (MBC) to yield prodigiosin | Aspartate racemase; KEGG: amino acid adenylation domain-containing protein | 0.456 |
| pigC | ESN64554.1 | Pyruvate, water dikinase, phosphoenolpyruvate--protein phosphotransferase; Involved in the biosynthesis of 2-methyl-3-n-amyl-pyrrole (MAP), one of the terminal products involved in the biosynthesis of the red antibiotic prodigiosin (Pig). Catalyzes the transfer of 2-methyl-3- n-amyl-pyrrole (MAP) to 4-methoxy-2,2'-bipyrrole-5-carbaldehyde (MBC) to yield prodigiosin | 6-deoxyerythronolide-B synthase, Phenylalanine racemase (ATP-hydrolyzing); KEGG: KR domain-containing protein | 0.459 |
| pigC | ESN64555.1 | Pyruvate, water dikinase, phosphoenolpyruvate--protein phosphotransferase; Involved in the biosynthesis of 2-methyl-3-n-amyl-pyrrole (MAP), one of the terminal products involved in the biosynthesis of the red antibiotic prodigiosin (Pig). Catalyzes the transfer of 2-methyl-3- n-amyl-pyrrole (MAP) to 4-methoxy-2,2'-bipyrrole-5-carbaldehyde (MBC) to yield prodigiosin | Phenylalanine racemase (atp-hydrolyzing); Belongs to the ATP-dependent AMP-binding enzyme family | 0.418 |
| pigC | ESN64556.1 | Pyruvate, water dikinase, phosphoenolpyruvate--protein phosphotransferase; Involved in the biosynthesis of 2-methyl-3-n-amyl-pyrrole (MAP), one of the terminal products involved in the biosynthesis of the red antibiotic prodigiosin (Pig). Catalyzes the transfer of 2-methyl-3- n-amyl-pyrrole (MAP) to 4-methoxy-2,2'-bipyrrole-5-carbaldehyde (MBC) to yield prodigiosin | Phenylalanine racemase (ATP-hydrolyzing); KEGG: amino acid adenylation protein | 0.472 |
| pigC | ESN64568.1 | Pyruvate, water dikinase, phosphoenolpyruvate--protein phosphotransferase; Involved in the biosynthesis of 2-methyl-3-n-amyl-pyrrole (MAP), one of the terminal products involved in the biosynthesis of the red antibiotic prodigiosin (Pig). Catalyzes the transfer of 2-methyl-3- n-amyl-pyrrole (MAP) to 4-methoxy-2,2'-bipyrrole-5-carbaldehyde (MBC) to yield prodigiosin | Formate C-acetyltransferase; KEGG: hypothetical protein | 0.812 |
| pigC | ESN64570.1 | Pyruvate, water dikinase, phosphoenolpyruvate--protein phosphotransferase; Involved in the biosynthesis of 2-methyl-3-n-amyl-pyrrole (MAP), one of the terminal products involved in the biosynthesis of the red antibiotic prodigiosin (Pig). Catalyzes the transfer of 2-methyl-3- n-amyl-pyrrole (MAP) to 4-methoxy-2,2'-bipyrrole-5-carbaldehyde (MBC) to yield prodigiosin | Transaldolase is important for the balance of metabolites in the pentose-phosphate pathway | 0.748 |
| pigC | ESN64573.1 | Pyruvate, water dikinase, phosphoenolpyruvate--protein phosphotransferase; Involved in the biosynthesis of 2-methyl-3-n-amyl-pyrrole (MAP), one of the terminal products involved in the biosynthesis of the red antibiotic prodigiosin (Pig). Catalyzes the transfer of 2-methyl-3- n-amyl-pyrrole (MAP) to 4-methoxy-2,2'-bipyrrole-5-carbaldehyde (MBC) to yield prodigiosin | 3-oxoacyl-(acyl-carrier-protein) reductase; KEGG: fabG, 3-oxoacyl-ACP reductase | 0.421 |
| pigC | ESN64609.1 | Pyruvate, water dikinase, phosphoenolpyruvate--protein phosphotransferase; Involved in the biosynthesis of 2-methyl-3-n-amyl-pyrrole (MAP), one of the terminal products involved in the biosynthesis of the red antibiotic prodigiosin (Pig). Catalyzes the transfer of 2-methyl-3- n-amyl-pyrrole (MAP) to 4-methoxy-2,2'-bipyrrole-5-carbaldehyde (MBC) to yield prodigiosin | Hypothetical protein; Uncharacterized protein; KEGG: mhpR, DNA-binding transcriptional activator MhpR | 0.644 |

| node1 | node2 | node1 annotation | node2 annotation | score |
| --- | --- | --- | --- | --- |
| pigC | ESN64612.1 | Pyruvate, water dikinase, phosphoenolpyruvate--protein phosphotransferase; Involved in the biosynthesis of 2-methyl-3-n-amyl-pyrrole (MAP), one of the terminal products involved in the biosynthesis of the red antibiotic prodigiosin (Pig). Catalyzes the transfer of 2-methyl-3- n-amyl-pyrrole (MAP) to 4-methoxy-2,2'-bipyrrole-5-carbaldehyde (MBC) to yield prodigiosin | 3-oxoacyl-(acyl-carrier-protein) reductase; KEGG: short-chain dehydrogenase/reductase SDR | 0.421 |
| pigC | ESN64620.1 | Pyruvate, water dikinase, phosphoenolpyruvate--protein phosphotransferase; Involved in the biosynthesis of 2-methyl-3-n-amyl-pyrrole (MAP), one of the terminal products involved in the biosynthesis of the red antibiotic prodigiosin (Pig). Catalyzes the transfer of 2-methyl-3- n-amyl-pyrrole (MAP) to 4-methoxy-2,2'-bipyrrole-5-carbaldehyde (MBC) to yield prodigiosin | Hypothetical protein; Uncharacterized protein; KEGG: hydrogenase 4 subunit H | 0.772 |
| pigC | ESN64654.1 | Pyruvate, water dikinase, phosphoenolpyruvate--protein phosphotransferase; Involved in the biosynthesis of 2-methyl-3-n-amyl-pyrrole (MAP), one of the terminal products involved in the biosynthesis of the red antibiotic prodigiosin (Pig). Catalyzes the transfer of 2-methyl-3- n-amyl-pyrrole (MAP) to 4-methoxy-2,2'-bipyrrole-5-carbaldehyde (MBC) to yield prodigiosin | Hypothetical protein; Belongs to the aldehyde dehydrogenase family | 0.627 |
| pigC | ESN64774.1 | Pyruvate, water dikinase, phosphoenolpyruvate--protein phosphotransferase; Involved in the biosynthesis of 2-methyl-3-n-amyl-pyrrole (MAP), one of the terminal products involved in the biosynthesis of the red antibiotic prodigiosin (Pig). Catalyzes the transfer of 2-methyl-3- n-amyl-pyrrole (MAP) to 4-methoxy-2,2'-bipyrrole-5-carbaldehyde (MBC) to yield prodigiosin | Hypothetical protein; This protein is a component of the acetyl coenzyme A carboxylase complex; first, biotin carboxylase catalyzes the carboxylation of the carrier protein and then the transcarboxylase transfers the carboxyl group to form malonyl-CoA | 0.864 |
| pigC | ESN64775.1 | Pyruvate, water dikinase, phosphoenolpyruvate--protein phosphotransferase; Involved in the biosynthesis of 2-methyl-3-n-amyl-pyrrole (MAP), one of the terminal products involved in the biosynthesis of the red antibiotic prodigiosin (Pig). Catalyzes the transfer of 2-methyl-3- n-amyl-pyrrole (MAP) to 4-methoxy-2,2'-bipyrrole-5-carbaldehyde (MBC) to yield prodigiosin | Pyruvate carboxylase; KEGG: acetyl-CoA carboxylase biotin carboxylase subunit | 0.824 |
| pigC | ESN64842.1 | Pyruvate, water dikinase, phosphoenolpyruvate--protein phosphotransferase; Involved in the biosynthesis of 2-methyl-3-n-amyl-pyrrole (MAP), one of the terminal products involved in the biosynthesis of the red antibiotic prodigiosin (Pig). Catalyzes the transfer of 2-methyl-3- n-amyl-pyrrole (MAP) to 4-methoxy-2,2'-bipyrrole-5-carbaldehyde (MBC) to yield prodigiosin | Unspecific monooxygenase; Belongs to the cytochrome P450 family | 0.474 |
| pigC | ESN64859.1 | Pyruvate, water dikinase, phosphoenolpyruvate--protein phosphotransferase; Involved in the biosynthesis of 2-methyl-3-n-amyl-pyrrole (MAP), one of the terminal products involved in the biosynthesis of the red antibiotic prodigiosin (Pig). Catalyzes the transfer of 2-methyl-3- n-amyl-pyrrole (MAP) to 4-methoxy-2,2'-bipyrrole-5-carbaldehyde (MBC) to yield prodigiosin | KEGG: hypothetical protein | 0.619 |
| pigC | ESN64983.1 | Pyruvate, water dikinase, phosphoenolpyruvate--protein phosphotransferase; Involved in the biosynthesis of 2-methyl-3-n-amyl-pyrrole (MAP), one of the terminal products involved in the biosynthesis of the red antibiotic prodigiosin (Pig). Catalyzes the transfer of 2-methyl-3- n-amyl-pyrrole (MAP) to 4-methoxy-2,2'-bipyrrole-5-carbaldehyde (MBC) to yield prodigiosin | annotation not available | 0.981 |
| pigC | ESN64984.1 | Pyruvate, water dikinase, phosphoenolpyruvate--protein phosphotransferase; Involved in the biosynthesis of 2-methyl-3-n-amyl-pyrrole (MAP), one of the terminal products involved in the biosynthesis of the red antibiotic prodigiosin (Pig). Catalyzes the transfer of 2-methyl-3- n-amyl-pyrrole (MAP) to 4-methoxy-2,2'-bipyrrole-5-carbaldehyde (MBC) to yield prodigiosin | KEGG: putative glutamate synthase (NADPH) small subunit | 0.827 |
| pigC | ESN64990.1 | Pyruvate, water dikinase, phosphoenolpyruvate--protein phosphotransferase; Involved in the biosynthesis of 2-methyl-3-n-amyl-pyrrole (MAP), one of the terminal products involved in the biosynthesis of the red antibiotic prodigiosin (Pig). Catalyzes the transfer of 2-methyl-3- n-amyl-pyrrole (MAP) to 4-methoxy-2,2'-bipyrrole-5-carbaldehyde (MBC) to yield prodigiosin | (+)-neomenthol dehydrogenase; Belongs to the short-chain dehydrogenases/reductases (SDR) family | 0.501 |
| pigC | ESN65033.1 | Pyruvate, water dikinase, phosphoenolpyruvate--protein phosphotransferase; Involved in the biosynthesis of 2-methyl-3-n-amyl-pyrrole (MAP), one of the terminal products involved in the biosynthesis of the red antibiotic prodigiosin (Pig). Catalyzes the transfer of 2-methyl-3- n-amyl-pyrrole (MAP) to 4-methoxy-2,2'-bipyrrole-5-carbaldehyde (MBC) to yield prodigiosin | 2-dehydro-3-deoxy-phosphogluconate aldolase; KEGG: 2-dehydro-3-deoxyphosphogluconate aldolase/4-hydroxy-2-oxoglutarate aldolase | 0.844 |
| pigC | ESN65061.1 | Pyruvate, water dikinase, phosphoenolpyruvate--protein phosphotransferase; Involved in the biosynthesis of 2-methyl-3-n-amyl-pyrrole (MAP), one of the terminal products involved in the biosynthesis of the red antibiotic prodigiosin (Pig). Catalyzes the transfer of 2-methyl-3- n-amyl-pyrrole (MAP) to 4-methoxy-2,2'-bipyrrole-5-carbaldehyde (MBC) to yield prodigiosin | Hypothetical protein; Uncharacterized protein; KEGG: aconitate hydratase domain-containing protein | 0.900 |
| pigC | ESN65067.1 | Pyruvate, water dikinase, phosphoenolpyruvate--protein phosphotransferase; Involved in the biosynthesis of 2-methyl-3-n-amyl-pyrrole (MAP), one of the terminal products involved in the biosynthesis of the red antibiotic prodigiosin (Pig). Catalyzes the transfer of 2-methyl-3- n-amyl-pyrrole (MAP) to 4-methoxy-2,2'-bipyrrole-5-carbaldehyde (MBC) to yield prodigiosin | 2-isopropylmalate synthase; Catalyzes the condensation of the acetyl group of acetyl-CoA with 3-methyl-2-oxobutanoate (2-oxoisovalerate) to form 3-carboxy-3- hydroxy-4-methylpentanoate (2-isopropylmalate) | 0.722 |
| pigC | ESN65136.1 | Pyruvate, water dikinase, phosphoenolpyruvate--protein phosphotransferase; Involved in the biosynthesis of 2-methyl-3-n-amyl-pyrrole (MAP), one of the terminal products involved in the biosynthesis of the red antibiotic prodigiosin (Pig). Catalyzes the transfer of 2-methyl-3- n-amyl-pyrrole (MAP) to 4-methoxy-2,2'-bipyrrole-5-carbaldehyde (MBC) to yield prodigiosin | Hypothetical protein; Uncharacterized protein; KEGG: kdgR, DNA-binding transcriptional regulator KdgR | 0.644 |
| pigC | ESN65216.1 | Pyruvate, water dikinase, phosphoenolpyruvate--protein phosphotransferase; Involved in the biosynthesis of 2-methyl-3-n-amyl-pyrrole (MAP), one of the terminal products involved in the biosynthesis of the red antibiotic prodigiosin (Pig). Catalyzes the transfer of 2-methyl-3- n-amyl-pyrrole (MAP) to 4-methoxy-2,2'-bipyrrole-5-carbaldehyde (MBC) to yield prodigiosin | Methionine synthase; Catalyzes the transfer of a methyl group from methyl- cobalamin to homocysteine, yielding enzyme-bound cob(I)alamin and methionine. Subsequently, remethylates the cofactor using methyltetrahydrofolate | 0.544 |
| pigC | ESN65217.1 | Pyruvate, water dikinase, phosphoenolpyruvate--protein phosphotransferase; Involved in the biosynthesis of 2-methyl-3-n-amyl-pyrrole (MAP), one of the terminal products involved in the biosynthesis of the red antibiotic prodigiosin (Pig). Catalyzes the transfer of 2-methyl-3- n-amyl-pyrrole (MAP) to 4-methoxy-2,2'-bipyrrole-5-carbaldehyde (MBC) to yield prodigiosin | Hypothetical protein; Uncharacterized protein; KEGG: IclR family transcriptional regulator | 0.644 |
| pigC | ESN65329.1 | Pyruvate, water dikinase, phosphoenolpyruvate--protein phosphotransferase; Involved in the biosynthesis of 2-methyl-3-n-amyl-pyrrole (MAP), one of the terminal products involved in the biosynthesis of the red antibiotic prodigiosin (Pig). Catalyzes the transfer of 2-methyl-3- n-amyl-pyrrole (MAP) to 4-methoxy-2,2'-bipyrrole-5-carbaldehyde (MBC) to yield prodigiosin | 3-oxoacyl-(acyl-carrier-protein) reductase; KEGG: short-chain dehydrogenase/reductase | 0.421 |
| pigC | ESN65396.1 | Pyruvate, water dikinase, phosphoenolpyruvate--protein phosphotransferase; Involved in the biosynthesis of 2-methyl-3-n-amyl-pyrrole (MAP), one of the terminal products involved in the biosynthesis of the red antibiotic prodigiosin (Pig). Catalyzes the transfer of 2-methyl-3- n-amyl-pyrrole (MAP) to 4-methoxy-2,2'-bipyrrole-5-carbaldehyde (MBC) to yield prodigiosin | Gluconate 5-dehydrogenase; Belongs to the short-chain dehydrogenases/reductases (SDR) family | 0.421 |
| pigC | ESN65397.1 | Pyruvate, water dikinase, phosphoenolpyruvate--protein phosphotransferase; Involved in the biosynthesis of 2-methyl-3-n-amyl-pyrrole (MAP), one of the terminal products involved in the biosynthesis of the red antibiotic prodigiosin (Pig). Catalyzes the transfer of 2-methyl-3- n-amyl-pyrrole (MAP) to 4-methoxy-2,2'-bipyrrole-5-carbaldehyde (MBC) to yield prodigiosin | KEGG: L-idonate 5-dehydrogenase | 0.669 |
| pigC | ESN65408.1 | Pyruvate, water dikinase, phosphoenolpyruvate--protein phosphotransferase; Involved in the biosynthesis of 2-methyl-3-n-amyl-pyrrole (MAP), one of the terminal products involved in the biosynthesis of the red antibiotic prodigiosin (Pig). Catalyzes the transfer of 2-methyl-3- n-amyl-pyrrole (MAP) to 4-methoxy-2,2'-bipyrrole-5-carbaldehyde (MBC) to yield prodigiosin | Hypothetical protein; Uncharacterized protein; KEGG: dehydrogenase | 0.421 |

| node1 | node2 | node1 annotation | node2 annotation | score |
| --- | --- | --- | --- | --- |
| pigC | ESN65409.1 | Pyruvate, water dikinase, phosphoenolpyruvate--protein phosphotransferase; Involved in the biosynthesis of 2-methyl-3-n-amyl-pyrrole (MAP), one of the terminal products involved in the biosynthesis of the red antibiotic prodigiosin (Pig). Catalyzes the transfer of 2-methyl-3- n-amyl-pyrrole (MAP) to 4-methoxy-2,2'-bipyrrole-5-carbaldehyde (MBC) to yield prodigiosin | Hypothetical protein; Uncharacterized protein; KEGG: acbL, cyclitol dehydrogenase | 0.669 |
| pigC | ESN65411.1 | Pyruvate, water dikinase, phosphoenolpyruvate--protein phosphotransferase; Involved in the biosynthesis of 2-methyl-3-n-amyl-pyrrole (MAP), one of the terminal products involved in the biosynthesis of the red antibiotic prodigiosin (Pig). Catalyzes the transfer of 2-methyl-3- n-amyl-pyrrole (MAP) to 4-methoxy-2,2'-bipyrrole-5-carbaldehyde (MBC) to yield prodigiosin | KEGG: 3-dehydroquinate synthase | 0.531 |
| pigC | ESN65453.1 | Pyruvate, water dikinase, phosphoenolpyruvate--protein phosphotransferase; Involved in the biosynthesis of 2-methyl-3-n-amyl-pyrrole (MAP), one of the terminal products involved in the biosynthesis of the red antibiotic prodigiosin (Pig). Catalyzes the transfer of 2-methyl-3- n-amyl-pyrrole (MAP) to 4-methoxy-2,2'-bipyrrole-5-carbaldehyde (MBC) to yield prodigiosin | KEGG: acetolactate synthase 2 catalytic subunit | 0.700 |
| pigC | aas | Pyruvate, water dikinase, phosphoenolpyruvate--protein phosphotransferase; Involved in the biosynthesis of 2-methyl-3-n-amyl-pyrrole (MAP), one of the terminal products involved in the biosynthesis of the red antibiotic prodigiosin (Pig). Catalyzes the transfer of 2-methyl-3- n-amyl-pyrrole (MAP) to 4-methoxy-2,2'-bipyrrole-5-carbaldehyde (MBC) to yield prodigiosin | Acyl-(acyl-carrier-protein)--phospholipid o-acyltransferase; Plays a role in lysophospholipid acylation. Transfers fatty acids to the 1-position via an enzyme-bound acyl-ACP intermediate in the presence of ATP and magnesium. Its physiological function is to regenerate phosphatidylethanolamine from 2-acyl-glycero-3- phosphoethanolamine (2-acyl-GPE) formed by transacylation reactions or degradation by phospholipase A1 | 0.622 |
| pigC | accC | Pyruvate, water dikinase, phosphoenolpyruvate--protein phosphotransferase; Involved in the biosynthesis of 2-methyl-3-n-amyl-pyrrole (MAP), one of the terminal products involved in the biosynthesis of the red antibiotic prodigiosin (Pig). Catalyzes the transfer of 2-methyl-3- n-amyl-pyrrole (MAP) to 4-methoxy-2,2'-bipyrrole-5-carbaldehyde (MBC) to yield prodigiosin | Acetyl-coa carboxylase; This protein is a component of the acetyl coenzyme A carboxylase complex; first, biotin carboxylase catalyzes the carboxylation of the carrier protein and then the transcarboxylase transfers the carboxyl group to form malonyl-CoA | 0.824 |
| pigC | aceE | Pyruvate, water dikinase, phosphoenolpyruvate--protein phosphotransferase; Involved in the biosynthesis of 2-methyl-3-n-amyl-pyrrole (MAP), one of the terminal products involved in the biosynthesis of the red antibiotic prodigiosin (Pig). Catalyzes the transfer of 2-methyl-3- n-amyl-pyrrole (MAP) to 4-methoxy-2,2'-bipyrrole-5-carbaldehyde (MBC) to yield prodigiosin | Pyruvate dehydrogenase (acetyl-transferring); Component of the pyruvate dehydrogenase (PDH) complex, that catalyzes the overall conversion of pyruvate to acetyl-CoA and CO(2) | 0.869 |
| pigC | aceF | Pyruvate, water dikinase, phosphoenolpyruvate--protein phosphotransferase; Involved in the biosynthesis of 2-methyl-3-n-amyl-pyrrole (MAP), one of the terminal products involved in the biosynthesis of the red antibiotic prodigiosin (Pig). Catalyzes the transfer of 2-methyl-3- n-amyl-pyrrole (MAP) to 4-methoxy-2,2'-bipyrrole-5-carbaldehyde (MBC) to yield prodigiosin | Dihydrolipoyllysine-residue acetyltransferase; The pyruvate dehydrogenase complex catalyzes the overall conversion of pyruvate to acetyl-CoA and CO(2) | 0.619 |
| pigC | ackA | Pyruvate, water dikinase, phosphoenolpyruvate--protein phosphotransferase; Involved in the biosynthesis of 2-methyl-3-n-amyl-pyrrole (MAP), one of the terminal products involved in the biosynthesis of the red antibiotic prodigiosin (Pig). Catalyzes the transfer of 2-methyl-3- n-amyl-pyrrole (MAP) to 4-methoxy-2,2'-bipyrrole-5-carbaldehyde (MBC) to yield prodigiosin | Acetate kinase; Catalyzes the formation of acetyl phosphate from acetate and ATP. Can also catalyze the reverse reaction | 0.463 |
| pigC | acnA | Pyruvate, water dikinase, phosphoenolpyruvate--protein phosphotransferase; Involved in the biosynthesis of 2-methyl-3-n-amyl-pyrrole (MAP), one of the terminal products involved in the biosynthesis of the red antibiotic prodigiosin (Pig). Catalyzes the transfer of 2-methyl-3- n-amyl-pyrrole (MAP) to 4-methoxy-2,2'-bipyrrole-5-carbaldehyde (MBC) to yield prodigiosin | Aconitate hydratase; Catalyzes the isomerization of citrate to isocitrate via cis- aconitate | 0.697 |
| pigC | adk | Pyruvate, water dikinase, phosphoenolpyruvate--protein phosphotransferase; Involved in the biosynthesis of 2-methyl-3-n-amyl-pyrrole (MAP), one of the terminal products involved in the biosynthesis of the red antibiotic prodigiosin (Pig). Catalyzes the transfer of 2-methyl-3- n-amyl-pyrrole (MAP) to 4-methoxy-2,2'-bipyrrole-5-carbaldehyde (MBC) to yield prodigiosin | Hypothetical protein; Catalyzes the reversible transfer of the terminal phosphate group between ATP and AMP. Plays an important role in cellular energy homeostasis and in adenine nucleotide metabolism | 0.757 |
| pigC | arnA | Pyruvate, water dikinase, phosphoenolpyruvate--protein phosphotransferase; Involved in the biosynthesis of 2-methyl-3-n-amyl-pyrrole (MAP), one of the terminal products involved in the biosynthesis of the red antibiotic prodigiosin (Pig). Catalyzes the transfer of 2-methyl-3- n-amyl-pyrrole (MAP) to 4-methoxy-2,2'-bipyrrole-5-carbaldehyde (MBC) to yield prodigiosin | Methionyl-trna formyltransferase, udp-glucuronate decarboxylase; Bifunctional enzyme that catalyzes the oxidative decarboxylation of UDP-glucuronic acid (UDP-GlcUA) to UDP-4-keto- arabinose (UDP-Ara4O) and the addition of a formyl group to UDP-4- amino-4-deoxy-L-arabinose (UDP-L-Ara4N) to form UDP-L-4-formamido- arabinose (UDP-L-Ara4FN). The modified arabinose is attached to lipid A and is required for resistance to polymyxin and cationic antimicrobial peptides | 0.723 |
| pigC | aroB | Pyruvate, water dikinase, phosphoenolpyruvate--protein phosphotransferase; Involved in the biosynthesis of 2-methyl-3-n-amyl-pyrrole (MAP), one of the terminal products involved in the biosynthesis of the red antibiotic prodigiosin (Pig). Catalyzes the transfer of 2-methyl-3- n-amyl-pyrrole (MAP) to 4-methoxy-2,2'-bipyrrole-5-carbaldehyde (MBC) to yield prodigiosin | 3-dehydroquinate synthase; Catalyzes the conversion of 3-deoxy-D-arabino-heptulosonate 7-phosphate (DAHP) to dehydroquinate (DHQ) | 0.531 |
| pigC | aroC | Pyruvate, water dikinase, phosphoenolpyruvate--protein phosphotransferase; Involved in the biosynthesis of 2-methyl-3-n-amyl-pyrrole (MAP), one of the terminal products involved in the biosynthesis of the red antibiotic prodigiosin (Pig). Catalyzes the transfer of 2-methyl-3- n-amyl-pyrrole (MAP) to 4-methoxy-2,2'-bipyrrole-5-carbaldehyde (MBC) to yield prodigiosin | Chorismate synthase; Catalyzes the anti-1,4-elimination of the C-3 phosphate and the C-6 proR hydrogen from 5-enolpyruvylshikimate-3-phosphate (EPSP) to yield chorismate, which is the branch point compound that serves as the starting substrate for the three terminal pathways of aromatic amino acid biosynthesis. This reaction introduces a second double bond into the aromatic ring system | 0.603 |
| pigC | astD | Pyruvate, water dikinase, phosphoenolpyruvate--protein phosphotransferase; Involved in the biosynthesis of 2-methyl-3-n-amyl-pyrrole (MAP), one of the terminal products involved in the biosynthesis of the red antibiotic prodigiosin (Pig). Catalyzes the transfer of 2-methyl-3- n-amyl-pyrrole (MAP) to 4-methoxy-2,2'-bipyrrole-5-carbaldehyde (MBC) to yield prodigiosin | Succinylglutamate-semialdehyde dehydrogenase; Catalyzes the NAD-dependent reduction of succinylglutamate semialdehyde into succinylglutamate | 0.627 |
| pigC | betA | Pyruvate, water dikinase, phosphoenolpyruvate--protein phosphotransferase; Involved in the biosynthesis of 2-methyl-3-n-amyl-pyrrole (MAP), one of the terminal products involved in the biosynthesis of the red antibiotic prodigiosin (Pig). Catalyzes the transfer of 2-methyl-3- n-amyl-pyrrole (MAP) to 4-methoxy-2,2'-bipyrrole-5-carbaldehyde (MBC) to yield prodigiosin | Choline dehydrogenase; Involved in the biosynthesis of the osmoprotectant glycine betaine. Catalyzes the oxidation of choline to betaine aldehyde and betaine aldehyde to glycine betaine at the same rate | 0.771 |
| pigC | betB | Pyruvate, water dikinase, phosphoenolpyruvate--protein phosphotransferase; Involved in the biosynthesis of 2-methyl-3-n-amyl-pyrrole (MAP), one of the terminal products involved in the biosynthesis of the red antibiotic prodigiosin (Pig). Catalyzes the transfer of 2-methyl-3- n-amyl-pyrrole (MAP) to 4-methoxy-2,2'-bipyrrole-5-carbaldehyde (MBC) to yield prodigiosin | Betaine-aldehyde dehydrogenase; Involved in the biosynthesis of the osmoprotectant glycine betaine. Catalyzes the reversible oxidation of betaine aldehyde to the corresponding acid | 0.627 |
| pigC | birA | Pyruvate, water dikinase, phosphoenolpyruvate--protein phosphotransferase; Involved in the biosynthesis of 2-methyl-3-n-amyl-pyrrole (MAP), one of the terminal products involved in the biosynthesis of the red antibiotic prodigiosin (Pig). Catalyzes the transfer of 2-methyl-3- n-amyl-pyrrole (MAP) to 4-methoxy-2,2'-bipyrrole-5-carbaldehyde (MBC) to yield prodigiosin | Biotin--(acetyl-coa-carboxylase) ligase; Acts both as a biotin--[acetyl-CoA-carboxylase] ligase and a biotin-operon repressor. In the presence of ATP, BirA activates biotin to form the BirA-biotinyl-5'-adenylate (BirA-bio-5'-AMP or holoBirA) complex. HoloBirA can either transfer the biotinyl moiety to the biotin carboxyl carrier protein (BCCP) subunit of acetyl-CoA carboxylase, or bind to the biotin operator site and inhibit transcription of the operon | 0.450 |
| pigC | cysC | Pyruvate, water dikinase, phosphoenolpyruvate--protein phosphotransferase; Involved in the biosynthesis of 2-methyl-3-n-amyl-pyrrole (MAP), one of the terminal products involved in the biosynthesis of the red antibiotic prodigiosin (Pig). Catalyzes the transfer of 2-methyl-3- n-amyl-pyrrole (MAP) to 4-methoxy-2,2'-bipyrrole-5-carbaldehyde (MBC) to yield prodigiosin | Adenylyl-sulfate kinase; Catalyzes the synthesis of activated sulfate | 0.784 |
| pigC | dld | Pyruvate, water dikinase, phosphoenolpyruvate--protein phosphotransferase; Involved in the biosynthesis of 2-methyl-3-n-amyl-pyrrole (MAP), one of the terminal products involved in the biosynthesis of the red antibiotic prodigiosin (Pig). Catalyzes the transfer of 2-methyl-3- n-amyl-pyrrole (MAP) to 4-methoxy-2,2'-bipyrrole-5-carbaldehyde (MBC) to yield prodigiosin | D-lactate dehydrogenase; Catalyzes the oxidation of D-lactate to pyruvate | 0.774 |
| pigC | dmpG | Pyruvate, water dikinase, phosphoenolpyruvate--protein phosphotransferase; Involved in the biosynthesis of 2-methyl-3-n-amyl-pyrrole (MAP), one of the terminal products involved in the biosynthesis of the red antibiotic prodigiosin (Pig). Catalyzes the transfer of 2-methyl-3- n-amyl-pyrrole (MAP) to 4-methoxy-2,2'-bipyrrole-5-carbaldehyde (MBC) to yield prodigiosin | 4-hydroxy-2-oxovalerate aldolase; Catalyzes the retro-aldol cleavage of 4-hydroxy-2- oxopentanoate to pyruvate and acetaldehyde. Is involved in the meta- cleavage pathway for the degradation of aromatic compounds | 0.722 |

| node1 | node2 | node1 annotation | node2 annotation | score |
| --- | --- | --- | --- | --- |
| pigC | eno | Pyruvate, water dikinase, phosphoenolpyruvate--protein phosphotransferase; Involved in the biosynthesis of 2-methyl-3-n-amyl-pyrrole (MAP), one of the terminal products involved in the biosynthesis of the red antibiotic prodigiosin (Pig). Catalyzes the transfer of 2-methyl-3- n-amyl-pyrrole (MAP) to 4-methoxy-2,2'-bipyrrole-5-carbaldehyde (MBC) to yield prodigiosin | Phosphopyruvate hydratase; Catalyzes the reversible conversion of 2-phosphoglycerate into phosphoenolpyruvate. It is essential for the degradation of carbohydrates via glycolysis | 0.983 |
| pigC | fsa | Pyruvate, water dikinase, phosphoenolpyruvate--protein phosphotransferase; Involved in the biosynthesis of 2-methyl-3-n-amyl-pyrrole (MAP), one of the terminal products involved in the biosynthesis of the red antibiotic prodigiosin (Pig). Catalyzes the transfer of 2-methyl-3- n-amyl-pyrrole (MAP) to 4-methoxy-2,2'-bipyrrole-5-carbaldehyde (MBC) to yield prodigiosin | Transaldolase is important for the balance of metabolites in the pentose-phosphate pathway | 0.748 |
| pigC | gabD | Pyruvate, water dikinase, phosphoenolpyruvate--protein phosphotransferase; Involved in the biosynthesis of 2-methyl-3-n-amyl-pyrrole (MAP), one of the terminal products involved in the biosynthesis of the red antibiotic prodigiosin (Pig). Catalyzes the transfer of 2-methyl-3- n-amyl-pyrrole (MAP) to 4-methoxy-2,2'-bipyrrole-5-carbaldehyde (MBC) to yield prodigiosin | Succinate-semialdehyde dehydrogenase; Belongs to the aldehyde dehydrogenase family | 0.627 |
| pigC | ghrB | Pyruvate, water dikinase, phosphoenolpyruvate--protein phosphotransferase; Involved in the biosynthesis of 2-methyl-3-n-amyl-pyrrole (MAP), one of the terminal products involved in the biosynthesis of the red antibiotic prodigiosin (Pig). Catalyzes the transfer of 2-methyl-3- n-amyl-pyrrole (MAP) to 4-methoxy-2,2'-bipyrrole-5-carbaldehyde (MBC) to yield prodigiosin | Gluconate 2-dehydrogenase; Catalyzes the NADPH-dependent reduction of glyoxylate and hydroxypyruvate into glycolate and glycerate, respectively | 0.736 |
| pigC | glpX | Pyruvate, water dikinase, phosphoenolpyruvate--protein phosphotransferase; Involved in the biosynthesis of 2-methyl-3-n-amyl-pyrrole (MAP), one of the terminal products involved in the biosynthesis of the red antibiotic prodigiosin (Pig). Catalyzes the transfer of 2-methyl-3- n-amyl-pyrrole (MAP) to 4-methoxy-2,2'-bipyrrole-5-carbaldehyde (MBC) to yield prodigiosin | Fructose-bisphosphatase; KEGG: glpX, Fructose-1,6-bisphosphatase, GlpX type | 0.743 |
| pigC | gpmA | Pyruvate, water dikinase, phosphoenolpyruvate--protein phosphotransferase; Involved in the biosynthesis of 2-methyl-3-n-amyl-pyrrole (MAP), one of the terminal products involved in the biosynthesis of the red antibiotic prodigiosin (Pig). Catalyzes the transfer of 2-methyl-3- n-amyl-pyrrole (MAP) to 4-methoxy-2,2'-bipyrrole-5-carbaldehyde (MBC) to yield prodigiosin | Bisphosphoglycerate mutase; Catalyzes the interconversion of 2-phosphoglycerate and 3- phosphoglycerate | 0.880 |
| pigC | gpmB | Pyruvate, water dikinase, phosphoenolpyruvate--protein phosphotransferase; Involved in the biosynthesis of 2-methyl-3-n-amyl-pyrrole (MAP), one of the terminal products involved in the biosynthesis of the red antibiotic prodigiosin (Pig). Catalyzes the transfer of 2-methyl-3- n-amyl-pyrrole (MAP) to 4-methoxy-2,2'-bipyrrole-5-carbaldehyde (MBC) to yield prodigiosin | Belongs to the phosphoglycerate mutase family. GpmB subfamily | 0.820 |
| pigC | guaA | Pyruvate, water dikinase, phosphoenolpyruvate--protein phosphotransferase; Involved in the biosynthesis of 2-methyl-3-n-amyl-pyrrole (MAP), one of the terminal products involved in the biosynthesis of the red antibiotic prodigiosin (Pig). Catalyzes the transfer of 2-methyl-3- n-amyl-pyrrole (MAP) to 4-methoxy-2,2'-bipyrrole-5-carbaldehyde (MBC) to yield prodigiosin | Gmp synthase (glutamine-hydrolyzing); Catalyzes the synthesis of GMP from XMP | 0.703 |
| pigC | ilvB | Pyruvate, water dikinase, phosphoenolpyruvate--protein phosphotransferase; Involved in the biosynthesis of 2-methyl-3-n-amyl-pyrrole (MAP), one of the terminal products involved in the biosynthesis of the red antibiotic prodigiosin (Pig). Catalyzes the transfer of 2-methyl-3- n-amyl-pyrrole (MAP) to 4-methoxy-2,2'-bipyrrole-5-carbaldehyde (MBC) to yield prodigiosin | KEGG: acetolactate synthase, large subunit, biosynthetic type | 0.700 |
| pigC | kduD | Pyruvate, water dikinase, phosphoenolpyruvate--protein phosphotransferase; Involved in the biosynthesis of 2-methyl-3-n-amyl-pyrrole (MAP), one of the terminal products involved in the biosynthesis of the red antibiotic prodigiosin (Pig). Catalyzes the transfer of 2-methyl-3- n-amyl-pyrrole (MAP) to 4-methoxy-2,2'-bipyrrole-5-carbaldehyde (MBC) to yield prodigiosin | KEGG: 2-deoxy-D-gluconate 3-dehydrogenase | 0.421 |
| pigC | lepA | Pyruvate, water dikinase, phosphoenolpyruvate--protein phosphotransferase; Involved in the biosynthesis of 2-methyl-3-n-amyl-pyrrole (MAP), one of the terminal products involved in the biosynthesis of the red antibiotic prodigiosin (Pig). Catalyzes the transfer of 2-methyl-3- n-amyl-pyrrole (MAP) to 4-methoxy-2,2'-bipyrrole-5-carbaldehyde (MBC) to yield prodigiosin | Hypothetical protein; Required for accurate and efficient protein synthesis under certain stress conditions. May act as a fidelity factor of the translation reaction, by catalyzing a one-codon backward translocation of tRNAs on improperly translocated ribosomes. Back-translocation proceeds from a post-translocation (POST) complex to a pre- translocation (PRE) complex, thus giving elongation factor G a second chance to translocate the tRNAs correctly. Binds to ribosomes in a GTP- dependent manner | 0.470 |
| pigC | leuA | Pyruvate, water dikinase, phosphoenolpyruvate--protein phosphotransferase; Involved in the biosynthesis of 2-methyl-3-n-amyl-pyrrole (MAP), one of the terminal products involved in the biosynthesis of the red antibiotic prodigiosin (Pig). Catalyzes the transfer of 2-methyl-3- n-amyl-pyrrole (MAP) to 4-methoxy-2,2'-bipyrrole-5-carbaldehyde (MBC) to yield prodigiosin | 2-isopropylmalate synthase; Catalyzes the condensation of the acetyl group of acetyl-CoA with 3-methyl-2-oxobutanoate (2-oxoisovalerate) to form 3-carboxy-3- hydroxy-4-methylpentanoate (2-isopropylmalate) | 0.722 |
| pigC | leuC | Pyruvate, water dikinase, phosphoenolpyruvate--protein phosphotransferase; Involved in the biosynthesis of 2-methyl-3-n-amyl-pyrrole (MAP), one of the terminal products involved in the biosynthesis of the red antibiotic prodigiosin (Pig). Catalyzes the transfer of 2-methyl-3- n-amyl-pyrrole (MAP) to 4-methoxy-2,2'-bipyrrole-5-carbaldehyde (MBC) to yield prodigiosin | Hypothetical protein; Catalyzes the isomerization between 2-isopropylmalate and 3- isopropylmalate, via the formation of 2-isopropylmaleate | 0.817 |
| pigC | leuD | Pyruvate, water dikinase, phosphoenolpyruvate--protein phosphotransferase; Involved in the biosynthesis of 2-methyl-3-n-amyl-pyrrole (MAP), one of the terminal products involved in the biosynthesis of the red antibiotic prodigiosin (Pig). Catalyzes the transfer of 2-methyl-3- n-amyl-pyrrole (MAP) to 4-methoxy-2,2'-bipyrrole-5-carbaldehyde (MBC) to yield prodigiosin | Hypothetical protein; Catalyzes the isomerization between 2-isopropylmalate and 3- isopropylmalate, via the formation of 2-isopropylmaleate | 0.816 |
| pigC | lldD | Pyruvate, water dikinase, phosphoenolpyruvate--protein phosphotransferase; Involved in the biosynthesis of 2-methyl-3-n-amyl-pyrrole (MAP), one of the terminal products involved in the biosynthesis of the red antibiotic prodigiosin (Pig). Catalyzes the transfer of 2-methyl-3- n-amyl-pyrrole (MAP) to 4-methoxy-2,2'-bipyrrole-5-carbaldehyde (MBC) to yield prodigiosin | L-lactate dehydrogenase (cytochrome); Catalyzes the conversion of L-lactate to pyruvate. Is coupled to the respiratory chain | 0.824 |
| pigC | lsrF | Pyruvate, water dikinase, phosphoenolpyruvate--protein phosphotransferase; Involved in the biosynthesis of 2-methyl-3-n-amyl-pyrrole (MAP), one of the terminal products involved in the biosynthesis of the red antibiotic prodigiosin (Pig). Catalyzes the transfer of 2-methyl-3- n-amyl-pyrrole (MAP) to 4-methoxy-2,2'-bipyrrole-5-carbaldehyde (MBC) to yield prodigiosin | Hypothetical protein; Involved in the degradation of phospho-AI-2, thereby terminating induction of the lsr operon and closing the AI-2 signaling cycle. Catalyzes the transfer of an acetyl moiety from 3-hydroxy-5- phosphonooxypentane-2,4-dione to CoA to form glycerone phosphate and acetyl-CoA | 0.871 |
| pigC | maeA | Pyruvate, water dikinase, phosphoenolpyruvate--protein phosphotransferase; Involved in the biosynthesis of 2-methyl-3-n-amyl-pyrrole (MAP), one of the terminal products involved in the biosynthesis of the red antibiotic prodigiosin (Pig). Catalyzes the transfer of 2-methyl-3- n-amyl-pyrrole (MAP) to 4-methoxy-2,2'-bipyrrole-5-carbaldehyde (MBC) to yield prodigiosin | Malate dehydrogenase (oxaloacetate-decarboxylating); NAD-dependent malic enzyme; KEGG: malate dehydrogenase | 0.989 |
| pigC | mdh | Pyruvate, water dikinase, phosphoenolpyruvate--protein phosphotransferase; Involved in the biosynthesis of 2-methyl-3-n-amyl-pyrrole (MAP), one of the terminal products involved in the biosynthesis of the red antibiotic prodigiosin (Pig). Catalyzes the transfer of 2-methyl-3- n-amyl-pyrrole (MAP) to 4-methoxy-2,2'-bipyrrole-5-carbaldehyde (MBC) to yield prodigiosin | Malate dehydrogenase; Catalyzes the reversible oxidation of malate to oxaloacetate | 0.925 |
| pigC | metG | Pyruvate, water dikinase, phosphoenolpyruvate--protein phosphotransferase; Involved in the biosynthesis of 2-methyl-3-n-amyl-pyrrole (MAP), one of the terminal products involved in the biosynthesis of the red antibiotic prodigiosin (Pig). Catalyzes the transfer of 2-methyl-3- n-amyl-pyrrole (MAP) to 4-methoxy-2,2'-bipyrrole-5-carbaldehyde (MBC) to yield prodigiosin | Methionine--trna ligase; Is required not only for elongation of protein synthesis but also for the initiation of all mRNA translation through initiator tRNA(fMet) aminoacylation | 0.746 |
| pigC | mmsA | Pyruvate, water dikinase, phosphoenolpyruvate--protein phosphotransferase; Involved in the biosynthesis of 2-methyl-3-n-amyl-pyrrole (MAP), one of the terminal products involved in the biosynthesis of the red antibiotic prodigiosin (Pig). Catalyzes the transfer of 2-methyl-3- n-amyl-pyrrole (MAP) to 4-methoxy-2,2'-bipyrrole-5-carbaldehyde (MBC) to yield prodigiosin | Methylmalonate-semialdehyde dehydrogenase (Acylating); KEGG: aldehyde dehydrogenase | 0.627 |

| node1 | node2 | node1 annotation | node2 annotation | score |
| --- | --- | --- | --- | --- |
| pigC | moaA | Pyruvate, water dikinase, phosphoenolpyruvate--protein phosphotransferase; Involved in the biosynthesis of 2-methyl-3-n-amyl-pyrrole (MAP), one of the terminal products involved in the biosynthesis of the red antibiotic prodigiosin (Pig). Catalyzes the transfer of 2-methyl-3- n-amyl-pyrrole (MAP) to 4-methoxy-2,2'-bipyrrole-5-carbaldehyde (MBC) to yield prodigiosin | Hypothetical protein; Catalyzes the cyclization of GTP to (8S)-3',8-cyclo-7,8- dihydroguanosine 5'-triphosphate | 0.666 |
| pigC | ndk | Pyruvate, water dikinase, phosphoenolpyruvate--protein phosphotransferase; Involved in the biosynthesis of 2-methyl-3-n-amyl-pyrrole (MAP), one of the terminal products involved in the biosynthesis of the red antibiotic prodigiosin (Pig). Catalyzes the transfer of 2-methyl-3- n-amyl-pyrrole (MAP) to 4-methoxy-2,2'-bipyrrole-5-carbaldehyde (MBC) to yield prodigiosin | Nucleoside-diphosphate kinase; Major role in the synthesis of nucleoside triphosphates other than ATP. The ATP gamma phosphate is transferred to the NDP beta phosphate via a ping-pong mechanism, using a phosphorylated active-site intermediate | 0.907 |
| pigC | nifJ | Pyruvate, water dikinase, phosphoenolpyruvate--protein phosphotransferase; Involved in the biosynthesis of 2-methyl-3-n-amyl-pyrrole (MAP), one of the terminal products involved in the biosynthesis of the red antibiotic prodigiosin (Pig). Catalyzes the transfer of 2-methyl-3- n-amyl-pyrrole (MAP) to 4-methoxy-2,2'-bipyrrole-5-carbaldehyde (MBC) to yield prodigiosin | annotation not available | 0.981 |
| pigC | nifV | Pyruvate, water dikinase, phosphoenolpyruvate--protein phosphotransferase; Involved in the biosynthesis of 2-methyl-3-n-amyl-pyrrole (MAP), one of the terminal products involved in the biosynthesis of the red antibiotic prodigiosin (Pig). Catalyzes the transfer of 2-methyl-3- n-amyl-pyrrole (MAP) to 4-methoxy-2,2'-bipyrrole-5-carbaldehyde (MBC) to yield prodigiosin | Belongs to the alpha-IPM synthase/homocitrate synthase family | 0.722 |
| pigC | nirD | Pyruvate, water dikinase, phosphoenolpyruvate--protein phosphotransferase; Involved in the biosynthesis of 2-methyl-3-n-amyl-pyrrole (MAP), one of the terminal products involved in the biosynthesis of the red antibiotic prodigiosin (Pig). Catalyzes the transfer of 2-methyl-3- n-amyl-pyrrole (MAP) to 4-methoxy-2,2'-bipyrrole-5-carbaldehyde (MBC) to yield prodigiosin | Ferredoxin--nad(+) reductase, nitrite reductase (nad(p)h); Belongs to the nitrite and sulfite reductase 4Fe-4S domain family | 0.770 |
| pigC | nuoC | Pyruvate, water dikinase, phosphoenolpyruvate--protein phosphotransferase; Involved in the biosynthesis of 2-methyl-3-n-amyl-pyrrole (MAP), one of the terminal products involved in the biosynthesis of the red antibiotic prodigiosin (Pig). Catalyzes the transfer of 2-methyl-3- n-amyl-pyrrole (MAP) to 4-methoxy-2,2'-bipyrrole-5-carbaldehyde (MBC) to yield prodigiosin | Hypothetical protein; NDH-1 shuttles electrons from NADH, via FMN and iron-sulfur (Fe-S) centers, to quinones in the respiratory chain. The immediate electron acceptor for the enzyme in this species is believed to be ubiquinone. Couples the redox reaction to proton translocation (for every two electrons transferred, four hydrogen ions are translocated across the cytoplasmic membrane), and thus conserves the redox energy in a proton gradient | 0.707 |
| pigC | nuoI | Pyruvate, water dikinase, phosphoenolpyruvate--protein phosphotransferase; Involved in the biosynthesis of 2-methyl-3-n-amyl-pyrrole (MAP), one of the terminal products involved in the biosynthesis of the red antibiotic prodigiosin (Pig). Catalyzes the transfer of 2-methyl-3- n-amyl-pyrrole (MAP) to 4-methoxy-2,2'-bipyrrole-5-carbaldehyde (MBC) to yield prodigiosin | Nadh dehydrogenase (quinone); NDH-1 shuttles electrons from NADH, via FMN and iron-sulfur (Fe-S) centers, to quinones in the respiratory chain. The immediate electron acceptor for the enzyme in this species is believed to be ubiquinone. Couples the redox reaction to proton translocation (for every two electrons transferred, four hydrogen ions are translocated across the cytoplasmic membrane), and thus conserves the redox energy in a proton gradient | 0.772 |
| pigC | patD | Pyruvate, water dikinase, phosphoenolpyruvate--protein phosphotransferase; Involved in the biosynthesis of 2-methyl-3-n-amyl-pyrrole (MAP), one of the terminal products involved in the biosynthesis of the red antibiotic prodigiosin (Pig). Catalyzes the transfer of 2-methyl-3- n-amyl-pyrrole (MAP) to 4-methoxy-2,2'-bipyrrole-5-carbaldehyde (MBC) to yield prodigiosin | Aminobutyraldehyde dehydrogenase; Belongs to the aldehyde dehydrogenase family | 0.627 |
| pigC | pckA | Pyruvate, water dikinase, phosphoenolpyruvate--protein phosphotransferase; Involved in the biosynthesis of 2-methyl-3-n-amyl-pyrrole (MAP), one of the terminal products involved in the biosynthesis of the red antibiotic prodigiosin (Pig). Catalyzes the transfer of 2-methyl-3- n-amyl-pyrrole (MAP) to 4-methoxy-2,2'-bipyrrole-5-carbaldehyde (MBC) to yield prodigiosin | Phosphoenolpyruvate carboxykinase (atp); Involved in the gluconeogenesis. Catalyzes the conversion of oxaloacetate (OAA) to phosphoenolpyruvate (PEP) through direct phosphoryl transfer between the nucleoside triphosphate and OAA | 0.914 |
| pigC | pfkA | Pyruvate, water dikinase, phosphoenolpyruvate--protein phosphotransferase; Involved in the biosynthesis of 2-methyl-3-n-amyl-pyrrole (MAP), one of the terminal products involved in the biosynthesis of the red antibiotic prodigiosin (Pig). Catalyzes the transfer of 2-methyl-3- n-amyl-pyrrole (MAP) to 4-methoxy-2,2'-bipyrrole-5-carbaldehyde (MBC) to yield prodigiosin | 6-phosphofructokinase; Catalyzes the phosphorylation of D-fructose 6-phosphate to fructose 1,6-bisphosphate by ATP, the first committing step of glycolysis | 0.797 |
| pigC | pflB | Pyruvate, water dikinase, phosphoenolpyruvate--protein phosphotransferase; Involved in the biosynthesis of 2-methyl-3-n-amyl-pyrrole (MAP), one of the terminal products involved in the biosynthesis of the red antibiotic prodigiosin (Pig). Catalyzes the transfer of 2-methyl-3- n-amyl-pyrrole (MAP) to 4-methoxy-2,2'-bipyrrole-5-carbaldehyde (MBC) to yield prodigiosin | Formate c-acetyltransferase; KEGG: formate acetyltransferase | 0.812 |
| pigC | pgi | Pyruvate, water dikinase, phosphoenolpyruvate--protein phosphotransferase; Involved in the biosynthesis of 2-methyl-3-n-amyl-pyrrole (MAP), one of the terminal products involved in the biosynthesis of the red antibiotic prodigiosin (Pig). Catalyzes the transfer of 2-methyl-3- n-amyl-pyrrole (MAP) to 4-methoxy-2,2'-bipyrrole-5-carbaldehyde (MBC) to yield prodigiosin | Glucose-6-phosphate isomerase; Belongs to the GPI family | 0.952 |
| pigC | pgk | Pyruvate, water dikinase, phosphoenolpyruvate--protein phosphotransferase; Involved in the biosynthesis of 2-methyl-3-n-amyl-pyrrole (MAP), one of the terminal products involved in the biosynthesis of the red antibiotic prodigiosin (Pig). Catalyzes the transfer of 2-methyl-3- n-amyl-pyrrole (MAP) to 4-methoxy-2,2'-bipyrrole-5-carbaldehyde (MBC) to yield prodigiosin | Belongs to the phosphoglycerate kinase family | 0.953 |
| pigC | pigA | Pyruvate, water dikinase, phosphoenolpyruvate--protein phosphotransferase; Involved in the biosynthesis of 2-methyl-3-n-amyl-pyrrole (MAP), one of the terminal products involved in the biosynthesis of the red antibiotic prodigiosin (Pig). Catalyzes the transfer of 2-methyl-3- n-amyl-pyrrole (MAP) to 4-methoxy-2,2'-bipyrrole-5-carbaldehyde (MBC) to yield prodigiosin | Butyryl-coa dehydrogenase; Involved in the biosynthesis of 4-methoxy-2,2'-bipyrrole-5- carbaldehyde (MBC), one of the terminal products involved in the biosynthesis of the red antibiotic prodigiosin (Pig). Catalyzes the desaturation of the L-prolyl-[PigG] to yield 1H-pyrrole-2-carbonyl- [PigG] | 0.845 |
| pigC | pigB | Pyruvate, water dikinase, phosphoenolpyruvate--protein phosphotransferase; Involved in the biosynthesis of 2-methyl-3-n-amyl-pyrrole (MAP), one of the terminal products involved in the biosynthesis of the red antibiotic prodigiosin (Pig). Catalyzes the transfer of 2-methyl-3- n-amyl-pyrrole (MAP) to 4-methoxy-2,2'-bipyrrole-5-carbaldehyde (MBC) to yield prodigiosin | Hypothetical protein; Involved in the biosynthesis of 2-methyl-3-n-amyl-pyrrole (MAP), one of the terminal products involved in the biosynthesis of the red antibiotic prodigiosin (Pig). Catalyzes the oxidation of dihydro form of MAP (H2MAP) to yield MAP | 0.970 |
| pigC | pigD | Pyruvate, water dikinase, phosphoenolpyruvate--protein phosphotransferase; Involved in the biosynthesis of 2-methyl-3-n-amyl-pyrrole (MAP), one of the terminal products involved in the biosynthesis of the red antibiotic prodigiosin (Pig). Catalyzes the transfer of 2-methyl-3- n-amyl-pyrrole (MAP) to 4-methoxy-2,2'-bipyrrole-5-carbaldehyde (MBC) to yield prodigiosin | Hypothetical protein; Involved in the biosynthesis of 2-methyl-3-n-amyl-pyrrole (MAP), one of the terminal products involved in the biosynthesis of the red antibiotic prodigiosin (Pig). Catalyzes the decarboxylation of pyruvate, followed by the modification of the resulting two-carbon fragment acetaldehyde at the C3 position of the 2-octenal (1,2-addition of acetaldehyde) giving 3-acetyloctanal | 0.854 |
| pigC | pigE | Pyruvate, water dikinase, phosphoenolpyruvate--protein phosphotransferase; Involved in the biosynthesis of 2-methyl-3-n-amyl-pyrrole (MAP), one of the terminal products involved in the biosynthesis of the red antibiotic prodigiosin (Pig). Catalyzes the transfer of 2-methyl-3- n-amyl-pyrrole (MAP) to 4-methoxy-2,2'-bipyrrole-5-carbaldehyde (MBC) to yield prodigiosin | Acetylornithine transaminase; Involved in the biosynthesis of 2-methyl-3-n-amyl-pyrrole (MAP), one of the terminal products involved in the biosynthesis of the red antibiotic prodigiosin (Pig). Catalyzes the transamination to the aldehyde group of 3-acetyloctanal, resulting in an aminoketone, which spontaneously cyclizes to yield the dihydro form of MAP (H2MAP) | 0.780 |
| pigC | pigF | Pyruvate, water dikinase, phosphoenolpyruvate--protein phosphotransferase; Involved in the biosynthesis of 2-methyl-3-n-amyl-pyrrole (MAP), one of the terminal products involved in the biosynthesis of the red antibiotic prodigiosin (Pig). Catalyzes the transfer of 2-methyl-3- n-amyl-pyrrole (MAP) to 4-methoxy-2,2'-bipyrrole-5-carbaldehyde (MBC) to yield prodigiosin | Hypothetical protein; Involved in the biosynthesis of 4-methoxy-2,2'-bipyrrole-5- carbaldehyde (MBC), one of the terminal products involved in the biosynthesis of the red antibiotic prodigiosin (Pig). Catalyzes the transfer of a methyl group from S-adenosyl-L-methionine (SAM) to the hydroxyl group of 4-hydroxy-2,2'-bipyrrole-5-carbaldehyde (HBC) to yield 4-methoxy-2,2'-bipyrrole-5-carbaldehyde (MBC) | 0.963 |
| pigC | pigG | Pyruvate, water dikinase, phosphoenolpyruvate--protein phosphotransferase; Involved in the biosynthesis of 2-methyl-3-n-amyl-pyrrole (MAP), one of the terminal products involved in the biosynthesis of the red antibiotic prodigiosin (Pig). Catalyzes the transfer of 2-methyl-3- n-amyl-pyrrole (MAP) to 4-methoxy-2,2'-bipyrrole-5-carbaldehyde (MBC) to yield prodigiosin | Hypothetical protein; Involved in the biosynthesis of 4-methoxy-2,2'-bipyrrole-5- carbaldehyde (MBC), one of the terminal products involved in the biosynthesis of the red antibiotic prodigiosin (Pig). Carrier of the L- prolyl group transferred from L-prolyl-AMP by PigI | 0.522 |
| pigC | pigH | Pyruvate, water dikinase, phosphoenolpyruvate--protein phosphotransferase; Involved in the biosynthesis of 2-methyl-3-n-amyl-pyrrole (MAP), one of the terminal products involved in the biosynthesis of the red antibiotic prodigiosin (Pig). Catalyzes the transfer of 2-methyl-3- n-amyl-pyrrole (MAP) to 4-methoxy-2,2'-bipyrrole-5-carbaldehyde (MBC) to yield prodigiosin | Glycine c-acetyltransferase; Involved in the biosynthesis of 4-methoxy-2,2'-bipyrrole-5- carbaldehyde (MBC), one of the terminal products involved in the biosynthesis of the red antibiotic prodigiosin (Pig). Carrier of the L- malonyl group (malonyl-S-PigH), which is decarboxylated by PigJ to yield a C2 carbanion acetyl-S-PigH. Then the pyrrolyl group of pyrrolyl-S-cysteinyl PigJ intermediate is captured by the C2 carbanion acetyl-S-PigH to yield the pyrrolyl-beta-ketoacyl-S-PigH. In the last step, PigH catalyzes the decarboxylative condensation between the pyrrolyl-beta-ketoacyl (pyrro [...] | 0.541 |

| node1 | node2 | node1 annotation | node2 annotation | score |
| --- | --- | --- | --- | --- |
| pigC | pigI | Pyruvate, water dikinase, phosphoenolpyruvate--protein phosphotransferase; Involved in the biosynthesis of 2-methyl-3-n-amyl-pyrrole (MAP), one of the terminal products involved in the biosynthesis of the red antibiotic prodigiosin (Pig). Catalyzes the transfer of 2-methyl-3- n-amyl-pyrrole (MAP) to 4-methoxy-2,2'-bipyrrole-5-carbaldehyde (MBC) to yield prodigiosin | D-alanine--poly(phosphoribitol) ligase; Involved in the biosynthesis of 4-methoxy-2,2'-bipyrrole-5- carbaldehyde (MBC), one of the terminal products involved in the biosynthesis of the red antibiotic prodigiosin (Pig) Catalyzes the conversion of L-proline to L-prolyl-AMP and the transfer of the L-prolyl group to acyl carrier protein PigG | 0.723 |
| pigC | pigJ | Pyruvate, water dikinase, phosphoenolpyruvate--protein phosphotransferase; Involved in the biosynthesis of 2-methyl-3-n-amyl-pyrrole (MAP), one of the terminal products involved in the biosynthesis of the red antibiotic prodigiosin (Pig). Catalyzes the transfer of 2-methyl-3- n-amyl-pyrrole (MAP) to 4-methoxy-2,2'-bipyrrole-5-carbaldehyde (MBC) to yield prodigiosin | Beta-ketoacyl-acyl-carrier-protein synthase i; Involved in the biosynthesis of 4-methoxy-2,2'-bipyrrole-5- carbaldehyde (MBC), one of the terminal products involved in the biosynthesis of the red antibiotic prodigiosin (Pig). Catalyzes the decarboxylation on the malonyl group attached to PigH to yield a C2 carbanion of acetyl-S-PigH. Then, the heterocyclic pyrrole group of PigG moves to the PigJ active site Cys-525 to generate a transient pyrrolyl-S-cysteinyl PigJ intermediate (acyl donor) whose pyrrolyl group is captured by the C2 carbanion of acetyl-S-PigH to yield the pyrrolyl-beta- [...] | 0.669 |
| pigC | pigM | Pyruvate, water dikinase, phosphoenolpyruvate--protein phosphotransferase; Involved in the biosynthesis of 2-methyl-3-n-amyl-pyrrole (MAP), one of the terminal products involved in the biosynthesis of the red antibiotic prodigiosin (Pig). Catalyzes the transfer of 2-methyl-3- n-amyl-pyrrole (MAP) to 4-methoxy-2,2'-bipyrrole-5-carbaldehyde (MBC) to yield prodigiosin | Hypothetical protein; Involved in the biosynthesis of 4-methoxy-2,2'-bipyrrole-5- carbaldehyde (MBC), one of the terminal products involved in the biosynthesis of the red antibiotic prodigiosin (Pig). Catalyzes the oxidation of the hydroxy group of 4-hydroxy-2,2'-bipyrrole-5-methanol (HBM) to yield 4-methoxy-2,2'-bipyrrole-5-carbaldehyde (MBC) | 0.778 |
| pigC | plsY | Pyruvate, water dikinase, phosphoenolpyruvate--protein phosphotransferase; Involved in the biosynthesis of 2-methyl-3-n-amyl-pyrrole (MAP), one of the terminal products involved in the biosynthesis of the red antibiotic prodigiosin (Pig). Catalyzes the transfer of 2-methyl-3- n-amyl-pyrrole (MAP) to 4-methoxy-2,2'-bipyrrole-5-carbaldehyde (MBC) to yield prodigiosin | Hypothetical protein; Catalyzes the transfer of an acyl group from acyl-phosphate (acyl-PO(4)) to glycerol-3-phosphate (G3P) to form lysophosphatidic acid (LPA). This enzyme utilizes acyl-phosphate as fatty acyl donor, but not acyl-CoA or acyl-ACP | 0.480 |
| pigC | ppc | Pyruvate, water dikinase, phosphoenolpyruvate--protein phosphotransferase; Involved in the biosynthesis of 2-methyl-3-n-amyl-pyrrole (MAP), one of the terminal products involved in the biosynthesis of the red antibiotic prodigiosin (Pig). Catalyzes the transfer of 2-methyl-3- n-amyl-pyrrole (MAP) to 4-methoxy-2,2'-bipyrrole-5-carbaldehyde (MBC) to yield prodigiosin | Phosphoenolpyruvate carboxylase; Forms oxaloacetate, a four-carbon dicarboxylic acid source for the tricarboxylic acid cycle | 0.887 |
| pigC | prmB | Pyruvate, water dikinase, phosphoenolpyruvate--protein phosphotransferase; Involved in the biosynthesis of 2-methyl-3-n-amyl-pyrrole (MAP), one of the terminal products involved in the biosynthesis of the red antibiotic prodigiosin (Pig). Catalyzes the transfer of 2-methyl-3- n-amyl-pyrrole (MAP) to 4-methoxy-2,2'-bipyrrole-5-carbaldehyde (MBC) to yield prodigiosin | Site-specific dna-methyltransferase (adenine-specific); Specifically methylates the 50S ribosomal protein L3 on a specific glutamine residue | 0.691 |
| pigC | prmC | Pyruvate, water dikinase, phosphoenolpyruvate--protein phosphotransferase; Involved in the biosynthesis of 2-methyl-3-n-amyl-pyrrole (MAP), one of the terminal products involved in the biosynthesis of the red antibiotic prodigiosin (Pig). Catalyzes the transfer of 2-methyl-3- n-amyl-pyrrole (MAP) to 4-methoxy-2,2'-bipyrrole-5-carbaldehyde (MBC) to yield prodigiosin | Hypothetical protein; Methylates the class 1 translation termination release factors RF1/PrfA and RF2/PrfB on the glutamine residue of the universally conserved GGQ motif | 0.691 |
| pigC | purL | Pyruvate, water dikinase, phosphoenolpyruvate--protein phosphotransferase; Involved in the biosynthesis of 2-methyl-3-n-amyl-pyrrole (MAP), one of the terminal products involved in the biosynthesis of the red antibiotic prodigiosin (Pig). Catalyzes the transfer of 2-methyl-3- n-amyl-pyrrole (MAP) to 4-methoxy-2,2'-bipyrrole-5-carbaldehyde (MBC) to yield prodigiosin | Phosphoribosylformylglycinamidine synthase involved in the purines biosynthetic pathway. Catalyzes the ATP-dependent conversion of formylglycinamide ribonucleotide (FGAR) and glutamine to yield formylglycinamidine ribonucleotide (FGAM) and glutamate | 0.552 |
| pigC | pyk | Pyruvate, water dikinase, phosphoenolpyruvate--protein phosphotransferase; Involved in the biosynthesis of 2-methyl-3-n-amyl-pyrrole (MAP), one of the terminal products involved in the biosynthesis of the red antibiotic prodigiosin (Pig). Catalyzes the transfer of 2-methyl-3- n-amyl-pyrrole (MAP) to 4-methoxy-2,2'-bipyrrole-5-carbaldehyde (MBC) to yield prodigiosin | Belongs to the pyruvate kinase family | 0.990 |
| pigC | rnhA | Pyruvate, water dikinase, phosphoenolpyruvate--protein phosphotransferase; Involved in the biosynthesis of 2-methyl-3-n-amyl-pyrrole (MAP), one of the terminal products involved in the biosynthesis of the red antibiotic prodigiosin (Pig). Catalyzes the transfer of 2-methyl-3- n-amyl-pyrrole (MAP) to 4-methoxy-2,2'-bipyrrole-5-carbaldehyde (MBC) to yield prodigiosin | Ribonuclease h; Endonuclease that specifically degrades the RNA of RNA-DNA hybrids | 0.805 |
| pigC | tal | Pyruvate, water dikinase, phosphoenolpyruvate--protein phosphotransferase; Involved in the biosynthesis of 2-methyl-3-n-amyl-pyrrole (MAP), one of the terminal products involved in the biosynthesis of the red antibiotic prodigiosin (Pig). Catalyzes the transfer of 2-methyl-3- n-amyl-pyrrole (MAP) to 4-methoxy-2,2'-bipyrrole-5-carbaldehyde (MBC) to yield prodigiosin | Hypothetical protein; Transaldolase is important for the balance of metabolites in the pentose-phosphate pathway | 0.748 |
| pigC | tdh | Pyruvate, water dikinase, phosphoenolpyruvate--protein phosphotransferase; Involved in the biosynthesis of 2-methyl-3-n-amyl-pyrrole (MAP), one of the terminal products involved in the biosynthesis of the red antibiotic prodigiosin (Pig). Catalyzes the transfer of 2-methyl-3- n-amyl-pyrrole (MAP) to 4-methoxy-2,2'-bipyrrole-5-carbaldehyde (MBC) to yield prodigiosin | L-threonine 3-dehydrogenase; Catalyzes the NAD(+)-dependent oxidation of L-threonine to 2- amino-3-ketobutyrate | 0.669 |
| pigC | tkt | Pyruvate, water dikinase, phosphoenolpyruvate--protein phosphotransferase; Involved in the biosynthesis of 2-methyl-3-n-amyl-pyrrole (MAP), one of the terminal products involved in the biosynthesis of the red antibiotic prodigiosin (Pig). Catalyzes the transfer of 2-methyl-3- n-amyl-pyrrole (MAP) to 4-methoxy-2,2'-bipyrrole-5-carbaldehyde (MBC) to yield prodigiosin | Transketolase; Catalyzes the transfer of a two-carbon ketol group from a ketose donor to an aldose acceptor, via a covalent intermediate with the cofactor thiamine pyrophosphate | 0.880 |
| pigC | tpiA | Pyruvate, water dikinase, phosphoenolpyruvate--protein phosphotransferase; Involved in the biosynthesis of 2-methyl-3-n-amyl-pyrrole (MAP), one of the terminal products involved in the biosynthesis of the red antibiotic prodigiosin (Pig). Catalyzes the transfer of 2-methyl-3- n-amyl-pyrrole (MAP) to 4-methoxy-2,2'-bipyrrole-5-carbaldehyde (MBC) to yield prodigiosin | Triose-phosphate isomerase; Involved in the gluconeogenesis. Catalyzes stereospecifically the conversion of dihydroxyacetone phosphate (DHAP) to D- glyceraldehyde-3-phosphate (G3P) | 0.713 |
| pigC | tyrA | Pyruvate, water dikinase, phosphoenolpyruvate--protein phosphotransferase; Involved in the biosynthesis of 2-methyl-3-n-amyl-pyrrole (MAP), one of the terminal products involved in the biosynthesis of the red antibiotic prodigiosin (Pig). Catalyzes the transfer of 2-methyl-3- n-amyl-pyrrole (MAP) to 4-methoxy-2,2'-bipyrrole-5-carbaldehyde (MBC) to yield prodigiosin | T-protein; KEGG: chorismate mutase | 0.519 |
| pigC | ubiA | Pyruvate, water dikinase, phosphoenolpyruvate--protein phosphotransferase; Involved in the biosynthesis of 2-methyl-3-n-amyl-pyrrole (MAP), one of the terminal products involved in the biosynthesis of the red antibiotic prodigiosin (Pig). Catalyzes the transfer of 2-methyl-3- n-amyl-pyrrole (MAP) to 4-methoxy-2,2'-bipyrrole-5-carbaldehyde (MBC) to yield prodigiosin | Hypothetical protein; Catalyzes the prenylation of para-hydroxybenzoate (PHB) with an all-trans polyprenyl group. Mediates the second step in the final reaction sequence of ubiquinone-8 (UQ-8) biosynthesis, which is the condensation of the polyisoprenoid side chain with PHB, generating the first membrane-bound Q intermediate 3-octaprenyl-4-hydroxybenzoate | 0.488 |
| pigC | uca | Pyruvate, water dikinase, phosphoenolpyruvate--protein phosphotransferase; Involved in the biosynthesis of 2-methyl-3-n-amyl-pyrrole (MAP), one of the terminal products involved in the biosynthesis of the red antibiotic prodigiosin (Pig). Catalyzes the transfer of 2-methyl-3- n-amyl-pyrrole (MAP) to 4-methoxy-2,2'-bipyrrole-5-carbaldehyde (MBC) to yield prodigiosin | KEGG: Urea carboxylase | 0.861 |
| pigC | zwf | Pyruvate, water dikinase, phosphoenolpyruvate--protein phosphotransferase; Involved in the biosynthesis of 2-methyl-3-n-amyl-pyrrole (MAP), one of the terminal products involved in the biosynthesis of the red antibiotic prodigiosin (Pig). Catalyzes the transfer of 2-methyl-3- n-amyl-pyrrole (MAP) to 4-methoxy-2,2'-bipyrrole-5-carbaldehyde (MBC) to yield prodigiosin | glucose-6-phosphate dehydrogenase; Catalyzes the oxidation of glucose 6-phosphate to 6- phosphogluconolactone | 0.884 |
