# Supplementary material for: Proteogenomic analysis of Serratia marcescens using computational subtractive genomics approach
Source: PLoS One. 2023 Apr 10;18(4):e0283993. doi: 10.1371/journal.pone.0283993 (PMC10085029; doi:10.1371/journal.pone.0283993)
Supplement: S3 Table — (DOCX) [file pone.0283993.s008.docx]

| S. No. | Strain | Accession | Proteome ID | Identity (%) |
| --- | --- | --- | --- | --- |
|  | FZSF02 | A0A6M5HS58 | UP000501174 | 99.3 |
|  | NCTC10211 | A0A380AMN7 | UP000254765 | 99.2 |
|  | B3R3 | A0A0N7JP81 | UP000058492 | 99.2 |
|  | S16 | A0A5C7CAG2 | UP000321126 | 99.1 |
|  | DSM 17174 | A0A7I0PX76 | UP000298282 | 99.1 |
|  | MSU-97 | A0A1Q4P508 | UP000185770 | 94 |
